# Design, synthesis and *in vitro* antiproliferative activity of new thiazolidinedione-1,3,4-oxadiazole hybrids as thymidylate synthase inhibitors

**DOI:** 10.1080/14756366.2020.1759581

**Published:** 2020-04-30

**Authors:** Zohor Mohammad Mahdi Alzhrani, Mohammad Mahboob Alam, Thikryat Neamatallah, Syed Nazreen

**Affiliations:** a Department of Chemistry, Faculty of Science, Albaha University, Albaha, Saudi Arabia; b Department of Pharmacology and Toxicology, Faculty of Pharmacy, King Abdulaziz University, Jeddah, Saudi Arabia

**Keywords:** 2,4-thiazolidinediones, 1,3,4-oxadiazoles, thymidylate synthase, molecular docking, pharmacokinetics

## Abstract

Thymidylate synthase (TS) has been an attention-grabbing area of research for the treatment of cancers due to their role in DNA biosynthesis. In the present study, we have synthesised a library of thiazolidinedione-1,3,4-oxadiazole hybrids as TS inhibitors. All the synthesised hybrids followed Lipinski and Veber rules which indicated good drug likeness properties upon oral administration. Among the synthesised hybrids, compound **9** and **10** displayed 4.5 and 4.4 folds activity of 5-Fluorouracil, respectively against MCF-7 cell line whereas 3.1 and 2.5 folds cytotoxicity against HCT-116 cell line. Furthermore, compound **9** and **10** also inhibited TS enzyme with IC_50_ = 1.67 and 2.21 µM, respectively. Finally, the docking studies of **9** and **10** were found to be consistent with *in vitro* TS results. From these studies, compound **9** and **10** has the potential to be developed as TS inhibitors.

## Introduction

Despite the availability of various chemotherapeutic agents, cancer is the second highest cause of deaths next to cardiovascular diseases.^1^^,^^2^ Thymidylate synthase (TS) has become an area of interest in cancer chemotherapy due to their important role in DNA biosynthesis.^3^^,^^4^ TS is responsible for the formation of deoxythymidine monophosphate (dTMP) from deoxyuridine monophosphate (dUMP) which is further phosphorylated to triphosphate group (dTTP), a direct precursor for DNA synthesis.^5–7^ TS inhibition causes inhibition of thymidylate biosynthesis which in turn causes cessation of cell growth and proliferation.^8^^,^^9^ Research on TS has been going on since many years, but still it is a challenge for medicinal chemists to develop new, safe and effective chemotherapeutic agents as TS inhibitor.

Thiazolidinediones (glitazones) are insulin sensitisers used for type II diabetes treatment. They are high-affinity ligands of PPAR-γ, which alleviates insulin resistance and effectively improves plasma glucose levels.^10–11^ Beside hypoglycaemic agents, thiazolidinedione derivatives are reported as anti-inflammatory,^12^ antimicrobial^13^ and anticancer agents.^14–16^ It has been mentioned that compounds which activate PPAR-γ might lead to differentiation induction of cancer cells.^17^ For example, a TZD analogue, efatutazone (CS-7017) is a potent PPAR-γ full agonist as well as a cancer differentiation-inducing agent.^18^ PPAR-γ agonists are reported to inhibit components of the insulin growth factor 1 (IGF1) pathway and modulate the activity of AMP-activated protein kinase (AMPK) pathway to reduce cancer risk.^19^^,^^20^ Thiazolidinediones derivatives have exerted anticancer effects by various mechanism of action *viz.* by inhibiting PI3K-α,^21^ blockade of the Raf/MEK/ERK and PI3K/Akt signalling pathways.^22^ On the other hand, 1,3,4-oxadiazoles are promising candidates in medicinal chemistry due to its wide applications. Zibotentan (ZD4054) containing 1,3,4-oxadiazole pharmacophore is an orally available selective antagonist of the ET-A receptor with potential antineoplastic effect for the treatment of various type of cancers.^23^^,^^24^ Recently, novel 1,3,4-oxadiazole bearing thioether derivatives has been reported as potential TS inhibitors.^25^

Combination of the important bioactive pharmacophores under one construct plays an important role in medicinal chemistry for the development of biologically active molecules with novel entity.^26^ To the best of our knowledge, anticancer effect of thiazolidinedione derivatives as TS inhibitors has not been reported till now. Therefore, we tried to conjugate thiazolidinedione and 1,3,4-oxadiazole under one construct to develop potential TS inhibitors. The present work describes the synthesis, *in silico* pharmacokinetic studies, *in vitro* antiproliferative and TS inhibitory activities. The docking studies have also been carried out for the most active compounds to understand the possible underlying molecular interactions.

## Experimental

### General

All the reagents and chemicals used in the present study were procured from Sigma Aldrich (Germany) and Loba (India). IR was recorded on Thermos scientific iS-50 by direct sampling method. NMR analysis was performed on Bruker 300 and 850 MHz instruments in either CDCl_3_ or DMSO-d_6_ solvents. Tetramethylsilane (TMS) was used as internal reference. Chemical shift and coupling constant are provided in Hertz and parts per million (ppm), respectively. Thermo scientific-LCQ Fleet (LCF10605) using electron spray ionisation method was used for recording the mass spectra and provided in m/z. Melting points were recorded on Stuart SMP40. Elemental analyses were performed on LEECO Elementar Elemental Analyser. The elemental analysis data were reported in % standard and found to be within ±0.4% of the calculated values. Purity of the compounds was checked on thin layer chromatography using silica gel G plate (Merck Germany). The spectral data and synthetic method of compounds **4–6** are provided in the Supplementary Material.

## Chemistry

### General procedure for the synthesis of thiazolidiene-2,4 dione-1,3,4-oxadiazole hybrids (7–21)

A mixture of compound **6** (0.01 mol) and different substituted aromatic hydrazides (0.01 mmol) in POCl_3_ (20 ml) was stirred and refluxed for 10–12 h. After reaction completion, the reaction mixture was poured onto crushed ice and neutralised with NaHCO_3_ solution. The resulting precipitate **7–21** was filtered, washed with excess cold water and dried. Purification of compounds **7–21** was done either by recrystallization in suitable solvents or by column chromatography using *n*-hexane and ethylacetate as eluents. The ^1^H NMR, ^13 ^C NMR and mass spectra of the compounds are provided in Supplementary Material.

**(*Z*)-5-(4-methoxybenzylidene)-3-((5-phenyl-1,3,4-oxadiazol-2-yl)methyl)thiazoli dine-2,4-dione (7).** Yield 70% mp 212–216 °C, ^1^H NMR (850 MHz, DMSO-d_6_) δ: 3.84 (s, 3H), 4.48 (s, 2H), 7.13 (d, 2H, *J* = 8.5 Hz), 7.44–7.47 (m, 2H), 7.61–7.73 (m, 5H), 7.96 (s, 1H); ^13^C NMR (213 MHz, DMSO-d_6_) δ: 42.63, 56.03, 115.53, 117.60, 125.16, 125.64, 127.08, 129.10, 129.98, 132.98, 134.62, 161.53, 161.89, 165.53, 167.26, 167.46; ESI + ve MS (*m/z*): 394 (M + H)^+^. Anal. Calc. for C_20_H_15_N_3_O_4_S: C, 61.06; H, 3.84; N, 10.68; O, 16.27; S, 8.15. Found: C, 61.05; H, 3.85; N, 10.66; O, 16.29; S, 8.14.

**(*Z*)-5-(4-methoxybenzylidene)-3-((5-(3-chlorophenyl)-1,3,4-oxadiazol-2-yl)methyl)thiazoli dine-2,4-dione (8).** Yield 60% mp 155–156 °C, ^1^H NMR (850 MHz, DMSO-d_6_) δ: 3.82 (s, 3H), 5.19 (s, 2H), 7.11 (d, 2H, *J* = 9.3 Hz), 7.61–7.64 (m, 3H), 7.70–7.71 (m, 1H), 7.92–7.95 (m, 2H), 7.96 (s, 1H); ^13^C NMR (213 MHz, DMSO-d_6_) δ: 35.78, 55.63, 115.15, 117.38, 125.05, 125.31, 125.45, 126.17, 131.67, 132.13, 132.56, 134.11, 134.13, 161.47, 161.65, 163.62, 164.97, 166.99; ESI + ve MS (*m/z*): 428 (M + H)^+^, 430 (M + 2 + H)^+^. Anal. Calc. for C_20_H_14_ClN_3_O_4_S: C, 56.14; H, 3.30; N, 9.82; O, 14.96; S, 7.49. Found: C, 56.11; H, 3.32; N, 9.83; O, 14.95; S, 7.50.

**(*Z*)-5-(4-methoxybenzylidene)-3-((5-(2-chlorophenyl)-1,3,4-oxadiazol-2-yl)methyl)thiazolid ine-2,4-dione (9).** Yield 65% mp 276–277, ^1^H NMR (300 MHz, DMSO-d_6_) δ: 3.84 (s, 3H), 4.40 (s, 2H), 7.13 (d, 2H, *J* = 8.7 Hz), 7.42–7.57 (m, 4H), 7.63 (d, 2H, *J* = 8.7 Hz), 7.95 (s, 1H); ^13^C NMR (75 MHz, DMSO-d_6_) δ: 40.80, 56.01, 115.51, 118.19, 125.93, 127.59, 129.71, 130.29, 132.83, 134.02, 161.32, 161.78, 164.93, 165.68, 167.52; ESI + ve MS (*m/z*): 428 (M + H)^+^, 430 (M + 2 + H)^+^.Anal. Calc. for C_20_H_14_ClN_3_O_4_S: C, 56.14; H, 3.30; N, 9.82; O, 14.96; S, 7.49. Found: C, 56.11; H, 3.32; N, 9.83; O, 14.95; S, 7.50.

**(*Z*)-5-(4-methoxybenzylidene)-3-((5-(4-bromophenyl)-1,3,4-oxadiazol-2-yl)methyl)thiazolid ine-2,4-dione (10).** Yield 65% mp 263–264 °C, ^1^H NMR (300 MHz, DMSO-d_6_) δ: 3.83 (s, 3H), 4.41 (s, 2H), 7.12 (d, 2H, *J* = 8.7 Hz), 7.63 (d, 2H, *J* = 8.4 Hz), 7.71 (d, 2H, *J* = 8.7 Hz), 7.80 (d, 2H, *J* = 8.4 Hz), 7.94 (s, 1H); ^13^C NMR (75 MHz, DMSO-d_6_) δ: 36.50, 56.01, 115.51, 120.59, 125.78, 129.98, 132.06, 132.83, 155.22, 161.98, 165.76, 166.42, 166.76; ESI + ve MS (*m/z*): 472 (M + H)^+^, 474 (M + 2 + H)^+^.Anal. Calc. for C_20_H_14_BrN_3_O_4_S: C, 50.86; H, 2.99; N, 8.90; O, 13.55; S, 6.79. Found: C, 50.84; H, 2.98; N, 8.92; O, 13.57; S, 6.81.

**(*Z*)-5-(4-methoxybenzylidene)-3-((5-*p*-tolyl-1,3,4-oxadiazol-2-yl)methyl)thiazolidine-2,4-dione (11).** Yield 72% mp 197–198 °C,^1^H NMR (300 MHz, DMSO-d_6_) δ: 2.38 (s, 3H), 3.83 (s, 3H), 5.19 (s, 2H), 7.12 (d, 2H, *J* = 8.7 Hz), 7.40 (d, 2H, *J* = 7.8 Hz), 7.62 (d, 2H, *J* = 8.7 Hz), 7.85 (d, 2H, *J* = 8.1 Hz), 7.97 (s, 1H); ^13^C NMR (75 MHz, DMSO-d_6_) δ: 21.58, 36.21, 56.01, 115.50, 117.73, 120.77, 125.70, 127.01, 130.45, 132.91, 134.52, 142.81, 161.37, 161.86, 165.17, 165.32, 167.30; ESI + ve MS (*m/z*): 408 (M + H)^+^. Anal. Calc. for C_21_H_17_N_3_O_4_S: C, 61.90; H, 4.21; N, 10.31; O, 15.71; S, 7.87. Found: C, 61.89; H, 4.20; N, 10.29; O, 15.72; S, 7.85.

**(*Z*)-5-(4-methoxybenzylidene)-3-((5-(2-hydroxyphenyl)-1,3,4-oxadiazol-2-yl)methyl)thiazol idine-2,4-dione (12).** Yield 65% mp 217–218 °C, ^1^H NMR (850 MHz, CDCl_3_) δ: 3.88 (s, 3H), 5.24 (s, 2H), 6.99–7.02 (m, 3H), 7.11 (d, 1H, *J* = 8.5 Hz), 7.44–7.46 (m, 1H), 7.49 (d, 2H, *J* = 8.5 Hz), 7.72 (dd, 1H, *J* = 9.3, 1.7 Hz), 7.95 (s, 1H), 9.94 (s, 1H); ^13^C NMR (213 MHz, CDCl_3_) δ: 35.55, 55.59, 107.60, 114.95, 117.11, 117.67, 120.01, 125.48, 126.76, 132.57, 134.09, 135.61, 157.63, 159.19, 161.92, 165.21, 165.29, 167.27; ESI + ve MS (m/z): 410 (M + H)^+^. Anal. Calc. for C_20_H_15_N_3_O_5_S: C, 58.67; H, 3.69; N, 10.26; O, 19.54; S, 7.83. Found: C, 58.65; H, 3.68; N, 10.27; O, 19.55; S, 7.85.

**(*Z*)-5-(4-methoxybenzylidene)-3-((5-*o*-tolyl-1,3,4-oxadiazol-2-yl)methyl)thiazolidine-2,4-dione (13).** Yield 57% mp 149–150 °C, ^1^H NMR (300 MHz, DMSO-d_6_) δ: 2.49 (s, 3H), 3.83 (s, 3H), 5.22 (s, 2H), 7.12 (d, 2H, *J* = 8.7 Hz), 7.56–7.72 (m, 4H), 7.92–7.98 (m, 3H); ^13^C NMR (75 MHz, DMSO-d_6_) δ: 21.55, 36.23, 56.01, 115.53, 117.72, 121.78, 125.79, 127.21, 128.38, 131.55, 132.94, 161.39, 161.90, 162.26, 165.38, 167.38; ESI + ve MS (*m/z*): 408 (M + H)^+^. Anal. Calc. for C_21_H_17_N_3_O_4_S: C, 61.90; H, 4.21; N, 10.31; O, 15.71; S, 7.87. Found: C, 61.89; H, 4.20; N, 10.29; O, 15.72; S, 7.85.

**(*Z*)-5-(4-methoxybenzylidene)-3-((5-(3-nitrophenyl)-1,3,4-oxadiazol-2-yl)methyl)thiazolidin e-2,4-dione (14).** Yield 75% mp 178–179 °C, ^1^H NMR (300 MHz, DMSO-d_6_) δ: 3.84 (s, 3H), 5.24 (s, 2H), 7.12 (d, 2H, *J* = 8.4 Hz), 7.63 (d, 2H, *J* = 8.4 Hz), 7.88–8.07 (m, 2H), 8.34–8.64 (m, 2H), 8.75 (s, 1H); ^13^C NMR (213 MHz, DMSO-d_6_) δ: 36.16, 56.03, 114.87, 115.54, 117.77, 121.63, 124.98, 125.69, 127.07, 131.93, 132.96, 133.20, 134.54, 148.68, 161.87, 162.33, 163.69, 165.36, 167.38; ESI + ve MS (*m/z*): 439 (M + H)^+^. Anal. Calc. for C_20_H_14_N_4_O_6_S: C, 54.79; H, 3.22; N, 12.78; O, 21.90; S, 7.31. Found: C, 54.80; H, 3.24; N, 12.75; O, 21.89; S, 7.32.

**(*Z*)-5-(4-methoxybenzylidene)-3-((5-*m*-tolyl-1,3,4-oxadiazol-2-yl)methyl)thiazolidine-2,4-dione (15).** Yield 65% mp 184–185 °C, ^1^H NMR (300 MHz, DMSO-d_6_) δ: 2.40 (s, 3H), 3.84 (s, 3H), 5.13 (s, 2H), 7.13 (d, 2H, *J* = 8.1 Hz), 7.38–7.51 (m, 2H), 7.62–7.69 (m, 2H), 7.76–7.96 (m, 2H), 7.98 (s, 1H); ^13^C NMR (75 MHz, DMSO-d_6_) δ: 21.27, 36.23, 56.02, 115.52, 117.75, 124.28, 125.71, 127.31, 129.85, 132.92, 133.31, 134.54, 139.44, 161.60, 161.87, 165.16, 167.35, 167.82; ESI + ve MS (*m/z*): 408 (M + H)^+^. Anal. Calc. for C_21_H_17_N_3_O_4_S: C, 61.90; H, 4.21; N, 10.31; O, 15.71; S, 7.87. Found: C, 61.89; H, 4.20; N, 10.29; O, 15.72; S, 7.85.

**(*Z*)-5-(4-methoxybenzylidene)-3-((5-(phenoxymethyl)-1,3,4-oxadiazol-2-yl)methyl)thiazolidine-2,4-dione (16).** Yield 70% mp 152–153 °C, ^1^H NMR (300 MHz, DMSO-d_6_) δ: 3.85 (s, 3H), 5.17 (s, 2H), 5.39 (s, 2H), 7.0–7.06 (m, 3H), 7.13 (d, 2H, *J* = 9.0 Hz), 7.32 (t, 2H, *J* = 6.9 Hz), 7.63 (d, 2H, *J* = 8.7 Hz), 7.97 (s, 1H); ^13^C NMR (75 MHz, DMSO-d_6_) δ: 36.14, 56.02, 59.87, 115.34, 115.54, 117.58, 122.28, 125.67, 130.09, 132.94, 134.71, 157.70, 161.92, 162.44, 164.17, 167.23; ESI + ve MS (*m/z*): 424 (M + H)^+^. Anal. Calc. for C_21_H_17_N_3_O_5_S: C, 59.57; H, 4.05; N, 9.92; O, 18.89; S, 7.57. Found: C, 59.59; H, 4.04; N, 9.91; O, 18.90; S, 7.53.

**(*Z*)-3-((5-((3-chlorophenoxy)methyl)-1,3,4-oxadiazol-2-yl)methyl)-5-(4-methoxybenzylidene)thiazolidine-2,4-dione (17).** Yield 72% mp 142–143 °C, ^1^H NMR (400 MHz, CDCl_3_) δ: 3.86 (s, 3H), 5.14 (s, 2H), 5.21 (s, 2H), 6.86–6.89 (m, 1H), 6.98–7.0 (m, 3H), 7.19–7.25 (m, 2H), 7.47 (d, 2H, *J* = 8.8 Hz), 7.91 (s, 1H); ^13^C NMR (100 MHz, CDCl_3_) δ: 35.63, 55.56, 59.90, 113.08, 114.87, 114.95, 115.69, 122.62, 125.54, 130.53, 132.38, 132.52, 135.20, 135.49, 158.07, 161.93, 162.87, 165.23, 167.19; ESI + ve MS (*m/z*): 458 (M + H)^+^, 460 (M + 2 + H)^+^. Anal. Calc. for C_21_H_16_ClN_3_O_5_S: C, 55.08; H, 3.52; N, 9.18; O, 17.47; S, 7.00. Found: C, 55.07; H, 3.54; N, 9.19; O, 17.45; S, 7.01.

**(*Z*)-3-((5-((2,3-dichlorophenoxy)methyl)-1,3,4-oxadiazol-2-yl)methyl)-5-(4-methoxybenzylidene)thiazolidine-2,4-dione (18).** Yield 72% mp 209–210 °C, ^1^H NMR (300 MHz, CDCl_3_) δ: 3.87 (s, 3H), 5.15 (s, 2H), 5.31 (s, 2H), 6.98–7.01 (m, 3H), 7.12–7.15 (m, 2H), 7.48 (d, 2H, *J* = 6.6 Hz), 7.91 (s, 1H); ^13^C NMR (100 MHz, CDCl_3_) δ: 35.64, 55.54, 61.23, 112.90, 114.96, 124.42, 125.53, 127.54, 132.52, 135.50, 155.69, 161.94, 162.58, 167.15, 168.32; ESI + ve MS (*m/z*): 492 (M + H)^+^, 494 (M + 2 + H)^+^. Anal. Calc. for C_21_H_15_Cl_2_N_3_O_5_S: C, 51.23; H, 3.07; N, 8.53; O, 16.25; S, 6.51. Found: C, 51.21; H, 3.06; N, 8.51; O, 16.27; S, 6.50.

**(*Z*)-5-(4-methoxybenzylidene)-3-((5-((naphthalen-1-yloxy)methyl)-1,3,4-oxadiazol-2-yl)methyl) thiazolidine-2,4-dione (19).** Yield 50% mp 220–221 °C, ^1^H NMR (300 MHz, CDCl_3_) δ: 3.86 (s, 3H), 5.15 (s, 2H), 5.43 (s, 2H), 6.93–7.01 (m, 3H), 7.33–7.51 (m, 7H), 7.76–7.79 (m, 1H), 7.90 (s, 1H); ^13^C NMR (213 MHz, CDCl_3_) δ: 41.43, 55.52, 62.18, 115.12, 116.65, 117.90, 124.26, 125.59, 125.65, 128.12, 128.38, 132.40, 132.49, 134.80, 135.37, 161.52, 164.29, 165.56, 167.35, 167.19; ESI + ve MS (*m/z*): 474 (M + H)^+^. Anal. Calc. for C_25_H_19_N_3_O_5_S: C, 63.41; H, 4.04; N, 8.87; O, 16.89; S, 6.77. Found: C, 63.42; H, 4.03; N, 8.90; O, 16.86; S, 6.78.

**(*Z*)-5-(4-methoxybenzylidene)-3-((5-((naphthalen-3-yloxy)methyl)-1,3,4-oxadiazol-2-yl)met hyl) thiazolidine-2,4-dione (20).** Yield 58% mp 210–211 °C, ^1^H NMR (400 MHz, CDCl_3_) δ: 3.86 (s, 3H), 5.15 (s, 2H), 5.35 (s, 2H), 6.98–7.0 (m, 3H), 7.18–7.20 (m, 2H), 7.46–7.48 (m, 4H), 7.74–7.76 (m, 2H), 7.89 (s, 1H); ^13^C NMR (213 MHz, CDCl_3_) δ: 41.45, 55.55, 62.21, 114.92, 116.64, 117.70, 123.93, 125.45, 125.65, 128.12, 128.38, 132.44, 132.56, 134.89, 135.46, 155.51, 161.71, 165.12, 166.19, 167.23, 167.64; ESI + ve MS (*m/z*): 474 (M + H)^+^. Anal. Calc. for C_25_H_19_N_3_O_5_S: C, 63.41; H, 4.04; N, 8.87; O, 16.89; S, 6.77. Found: C, 63.42; H, 4.03; N, 8.90; O, 16.86; S, 6.78.

**(*Z*)-5-(4-methoxybenzylidene)-3-((5-((quinolin-8-yloxy)methyl)-1,3,4-oxadiazol-2-yl)methyl) thiazolidine-2,4-dione (21).** Yield 70% mp 161–162 °C, ^1^H NMR (300 MHz, DMSO-d_6_) δ: 3.84 (s, 3H), 5.19 (s, 2H), 5.62 (s, 2H), 7.11–7.14 (m, 2H), 7.36 (d, 1H, *J* = 7.8 Hz), 7.46–7.64 (m, 5H), 7.96 (s, 1H); 8.33 (d, 1H, *J* = 9.3 Hz), 8.85–8.86 (m, 1H); ^13^C NMR (75 MHz, DMSO-d_6_) δ: 35.69, 58.53, 60.48, 111.46, 115.04, 117.05, 121.07, 121.25, 125.80, 128.53, 129.42, 132.45, 134.23, 138.45, 138.87, 149.32, 152.58, 160.47, 162.44, 163.32, 163.73, 166.72; ESI + ve MS (*m/z*): 475 (M + H)^+^. Anal. Calc. for C_24_H_18_N_4_O_5_S: C, 60.75; H, 3.82; N, 11.81; O, 16.86; S, 6.76. Found: C, 60.74; H, 3.81; N, 11.80; O, 16.87; S, 6.77.

## Cytotoxicity activity

The methods are provided in Supplementary material.

### *In vitro* thymidylate synthase assay

TS activity was performed according to the method as described by Wahab and Friedkin^27^ with a slight modification according to Santi.^28^ (Refer to Supplementary material for the method.)

## Molecular docking

**Figure 1.**
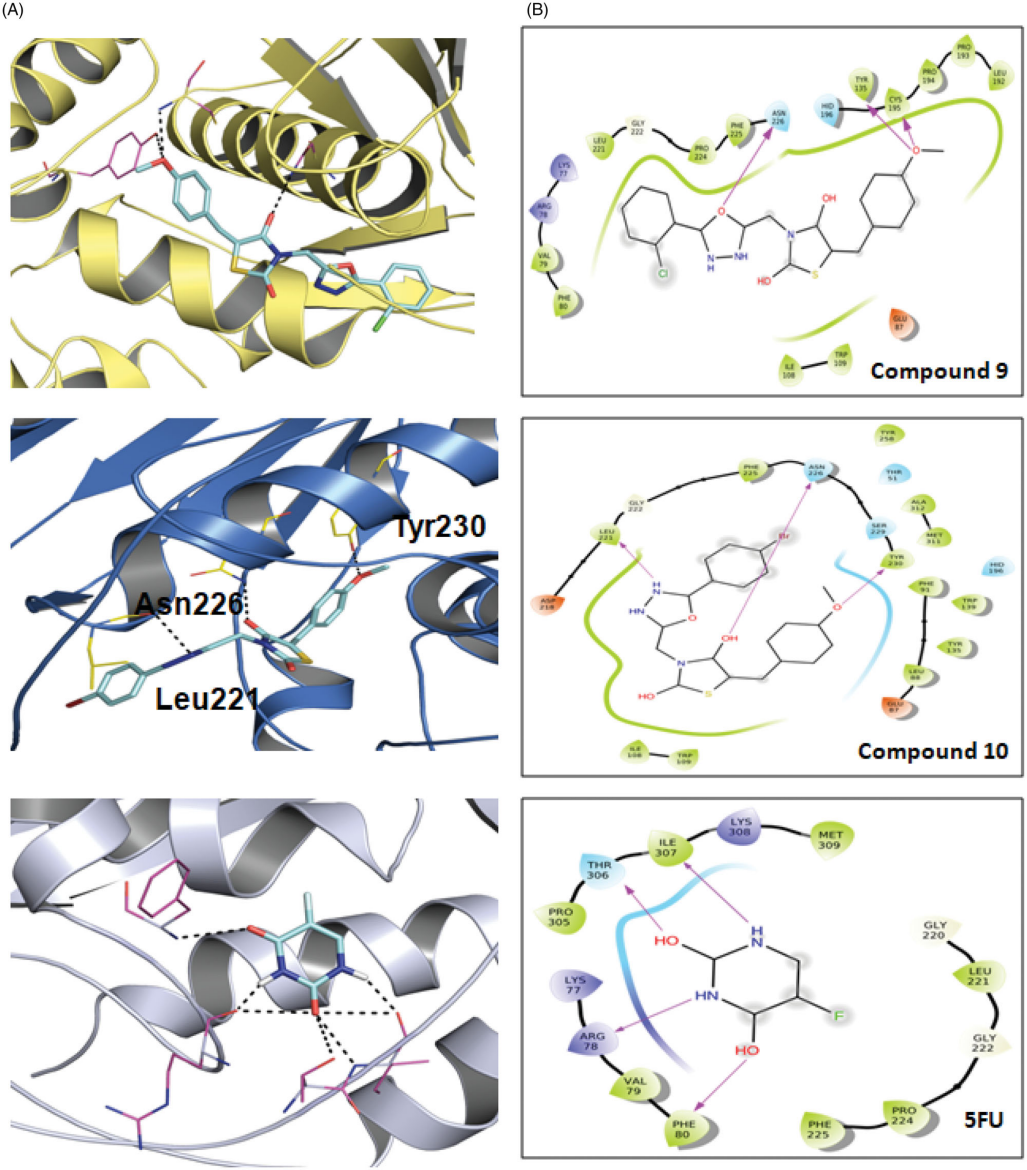
Molecular docking of the active compounds **9, 10** and **5FU** against Thimidylate synthase (TS) protein 6QXG. A: Binding mode of **9, 10** and **5FU** at TS binding site 3D plot. B: Binding mode of **9, 10** and **5FU** at TS binding site 2D plot. 5FU: -5-fluorouracil.

Docking studies were performed at Intel(R) Core(TM) i3 CPU(2.3 GHz) with XP-based operating system (Windows 2007). 2 D structures of the compounds were drawn by Marvin Sketch and then converted into 3 D structures and saved in pdb file format. Ligand preparation was done by assigning Gastegier charges, merging non-polar hydrogen’s, and saving in PDBQT file format using AutoDock Tools (ADT) 1.5.4. X-ray crystal structure of DNA (PDB ID: 6QXG) was obtained from the Protein Data Bank (http://www.rcsb.org/pdb). Gastegier charges were assigned to DNA and saved in PDBQT file format using ADT. Preparation of parameter files for grid and docking was done using ADT. Docking was performed on AutoDock 4.0 (Scripps Research Institute, USA) considering all the rotatable bonds of the ligands as rotatable and DNA as rigid.^29^ The grid centre was established by centring the grid box on whole DNA. Grid boxsize of 60 × 80 × 110 Å with 0.375 Å spacing was used. Macromolecule docking was performed using an empirical-free energy function and Lamarckian Genetic Algorithm, with an initial population of 150 randomly placed individuals, a maximum number of 2,500,000 energy evaluations, a mutation rate of 0.02, and cross-over rate of 0.80. Fifty independent docking runs were performed of each ligand and DNA–ligand complex for lowest free energy of binding conformation from the largest cluster, which was written and saved in PDBQT format. The results are summarised in Table 4 and Figure 1.

## Results and discussion

### Chemistry

**Scheme 1.**
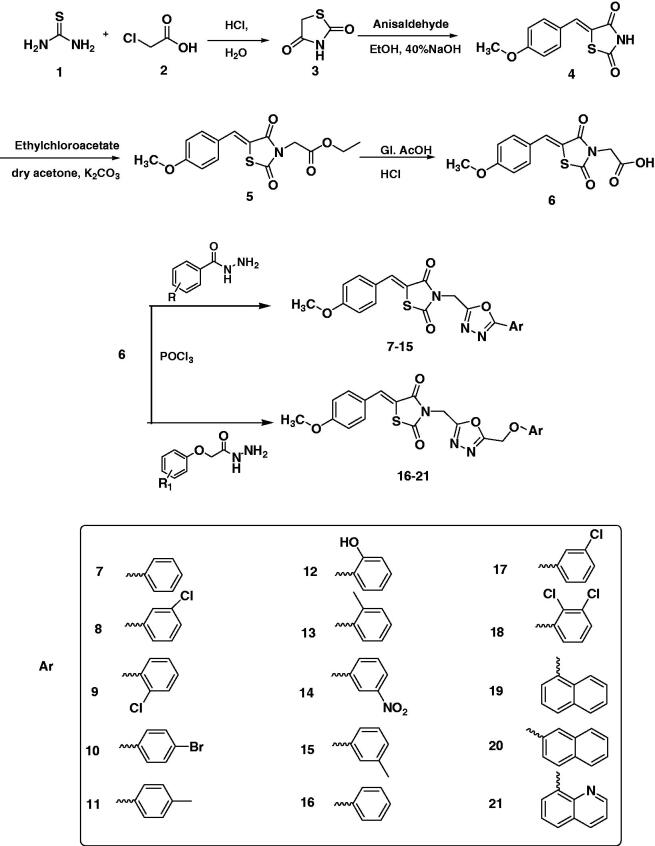
Synthesis of thiazolidinedione-1,3,4-oxadiazole hybrids.

Thiazolidinediones **3** was prepared by refluxing thiourea and chloroacetic acid in hydrochloric acid and water which on Knovenagel condensation with anisaldehyde in ethanol and aqueous sodium hydroxide gives intermediate benzylidene thiazolidinediones **4.** Alkylation of intermediate **4** with ethyl chloroacetate in the presence of anhydrous potassium carbonate and dry acetone followed by acidic hydrolysis using glacial acetic acid and hydrochloric acid gives the key intermediate **6.** The key intermediate **6** was then refluxed with different aromatic acid hydrazides and substituted phenoxy acetic acid hydrazides in the presence of phosphorus oxychloride to give the target compounds **7–15** and **16–21,** respectively in moderate to good yield (Scheme 1). The structures of the synthesised hybrids were confirmed by different analytical techniques such as ^1^H NMR, ^13^C NMR, IR spectroscopy, elemental analyser and mass spectrometry. Formation of the condensed product **4** was supported by the presence of two doublets at δ 7.09 (*J* = 8.7 Hz) and δ 7.56 (*J* = 8.7 Hz) which was assigned to four aromatic protons, a singlet at δ 7.75 for olefinic protons bridging phenyl and thiazolidinedione rings and δ 11.92 for NH of TZD ring in the ^1^H NMR. This structure was supported by ^13 ^C NMR which revealed the downfield signals at δ 161.45 for olefinic C=C and two signals at δ 167.88 and δ 168.40 for carbonyl groups of TZD ring. The NH and C=O bands of compound **4** were observed at 3215 cm^−1^(broad) and 1728 and 1690 cm^−1^, respectively in the IR spectrum. The conversion of compound **4** to compound **5** was observed by the disappearance of NH signal at δ 11.92 and appearance of a singlet at δ 4.47 for N–CH_2_, quartette at δ 4.17 (*J* = 7.2, 14.1 Hz) for O–CH_2_ and a triplet at δ 1.21 (*J* = 6.9 Hz) for terminal methyl protons of the ester in the ^1^H NMR whereas in ^13^C NMR these signals appeared at δ 42.60, δ 62.15 and δ 14.38 for N–CH_2,_ O–CH_2_ and terminal CH_3,_ respectively. The absence of quartet and triplet and presence of broad signal at δ 13.44 in the ^1^H NMR of key intermediate **6** supported its formation. This data was further supported by ^13 ^C NMR exhibiting one additional downfield signal for new carbonyl group of the acid and IR spectrum exhibited broad signal for hydroxyl proton at 3014 cm^−1^. Compound **6** was finally confirmed by the appearance of molecular ion peak at 292 (M − H)^+^ in mass spectrometry. The formation of the target compounds **7–21** was confirmed by the presence of additional aromatic protons in the range of δ 6.99–8.86 in their ^1^H NMR spectra. The absence of carboxyl proton signal in the target compounds **7–21** which was present in the ^1^H NMR spectrum of the key intermediate **6** also support their formation. Further structural confirmation of the target compounds **7–21** was provided by the presence of absorption bands in the range of 1509–1589 cm^−1^ for C=N stretching of oxadiazole ring in the IR spectra and the signals in the range of δ 159.19–166.19 for C=N of oxadiazole carbons in the ^13 ^C NMR spectra of these compounds. The target compounds **16–21** exhibited additional signal as singlet in the range of δ 5.21–5.62 for O–CH_2_ bridged methylene aromatic ring and 1,3,4-oxadiazole ring. Finally, confirmatory evidence was done by their mass spectral data.

### Pharmacokinetics studies/absorption, distribution, metabolism elimination (ADME) predictions

The capability of a drug to exhibit a pharmacological or therapeutic effect depends upon various physiochemical properties of the drug. Nowadays, ADME predictions of molecules are studied in initial phases of drug development by *in silico* approaches to generate potential lead molecules. Drug-likeness in drug development gives an idea whether the particular synthesised compound is comparable to already existing drug molecules in the market. The synthesised molecule expected to be an orally active drug should obey Lipinski rule^30^ which states the following four criteria: molecular weight should not be more than 500, hydrogen bond acceptor should not be more than 10, hydrogen bond donor should not be more than 5 and partition coefficient (Clog P) should not be more than 5. Violation of any of these criteria would result in problems of bioavailability upon oral administration. According to Veber et al.,^31^ number of rotatable bonds should be ≤10 which is an indicator for good bioavailability. In the present study, we calculated several parameters for predicting drug-likeness properties of synthesised compounds. The thiazolidinedione linked 1,3,4-oxadiazole hybrids (**7–21**) were subjected to following *in silico* physicochemical studies: number of rotatable bonds (nROTB), hydrogen bond acceptor (HBA), hydrogen bond donor (HBD), lipophilicity (iLogP) and topological polar surface area (TPSA). *In silico* %age absorption were calculated using the reported formula [(%ABS = 109–(0.345 X TPSA)]. From the results, it was observed that % absorption was found to be in the range of 54.98–70.76%. Compound **7, 8, 9, 10, 11,** and **13** showed highest *in silico* % absorption 70.76% whereas compound **14** revealed 54.98 *in silico* % absorption. All the synthesised compounds follow Lipinski rule of 5. Molecular weight ranges from 394 to 492 (<500), HBA range 6–8 (≤10), HBD range 0–1 (≤5), iLogP (lipophilicity) was found to be in the range 2.87–3.84 (≤5) which suggest that these compounds upon administration possess good drug likeness properties (Table 1). All the compounds except **14** and **21** exhibited high gastro-intestinal absorption and they are impermeable to brain. Furthermore, all the compounds showed nROTB in the range 5–7 (<10) suggesting good bioavailability. All the tested compounds except **14** revealed TPSA range 110.83–132.95 Å^2^ (<140 Å^2^), which indicates good intestinal absorption. From these parameters, these synthesised compounds exhibited good drug likeness properties.

**Table 1.** Pharmacokinetics/ADME predictions of the target compounds **7–21.**

No.	Lipinski parameters					nROTB^e^	TPSA^f^	% ABS^g^	BBB^h^	GI ABS^i^
	MW^a^	HBA^b^	HBD^c^	iLogP^d^	Violations					
7	394	6	0	3.3	0	5	110.83	70.76	No	High
8	430	6	0	3.62	0	5	110.83	70.76	No	High
9	430	6	0	3.5	0	5	110.83	70.76	No	High
10	474	6	0	3.69	0	5	110.83	70.76	No	High
11	408	6	0	3.6	0	5	110.83	70.76	No	High
12	410	7	1	3.21	0	5	131.06	63.78	No	High
13	408	6	0	3.46	0	5	110.83	70.76	No	High
14	439	8	0	2.87	0	6	156.65	54.98	No	Low
15	408	6	0	3.79	0	5	110.83	70.76	No	High
16	424	7	0	3.39	0	7	120.06	67.60	No	High
17	460	7	0	3.81	0	7	120.06	67.60	No	High
18	494	7	0	3.17	0	7	120.06	67.60	No	High
19	474	7	0	3.74	0	7	120.06	67.60	No	High
20	474	7	0	3.84	0	7	120.06	67.60	No	High
21	475	8	0	3.22	0	7	132.95	63.13	No	Low

^a^Molecular weight.

^b^Hydrogen bond acceptor.

^c^Hydrogen bond donor.

^d^ Partition coefficient.

^e^Number of rotatable bonds.

^f^Topological polar surface area.

^g^Absorption %.

^h^Blood brain barrier.

^i^Gastro-intestinal absorption.

### Biological evaluation

#### Cytotoxicity assay

The newly synthesised compounds **7–21** were tested for their cytotoxicity against human cancer cell line *viz*. breast and colorectal using MTT assay. The IC_50_ value of the synthesised compounds is presented in Table 2. From the results, it has been observed that the synthesised compounds showed significant to moderate growth inhibition compared to the reference drug, 5-fluorouracil (5FU). The cytotoxicity results revealed that compound **9**, **10** and **15** showed promising activity. Compound **9** and **10** showed significant cytotoxicity with IC_50_ = 7.47 µM and IC_50_ = 7.87 µM, respectively against MCF-7 whereas reference drug 5-FU exhibited IC_50_ = 34.82 µM. Compound **9** (IC_50_ = 7.47 µM) and **10** (IC_50_ = 7.87 µM) displayed 4.5 and 4.4 folds activity of the 5-FU, respectively against MCF-7 cell line. Against HCT-116 cell line, four compounds **9**, **10**, **14** and **15** showed excellent cytotoxicity with IC_50_ value of 12.84, 16.14, 42.21 and 39.60 µM, respectively whereas reference drug 5-FU exhibited IC_50_ = 40.51 µM. It was noticed that the same compound **9** and **10** showed 3.1 and 2.5 folds cytotoxicity compared to5-FU against HCT-116 while compound **14** and **15** was found to be equipotent to the reference drug with IC_50_ = 42.2 µM and IC_50_ = 39.60 µM, respectively. The moderate cytotoxicity was observed by compounds **7**, **8**, **11**, **12**, **13**, **16**, **17**, **19** and **21** with IC_50_ in the range 43.37–65.17 µM and 55.5–69.50 µM against MCF-7 and HCT-116, respectively while compound **18** and **20** were found to be inactive (IC_50_ > 100) against the tested cell lines. Among the synthesised compounds, compound **9** and **10** were the most potent against both MCF-7 and HCT-116 cell line. From the cytotoxicity results, it was noted that the 1,3,4-oxadiazole-thiazolidienedione hybrids derived from substituted aromatic acids hydrazides (**7–15**) were found to exhibit significant cytotoxicity (IC_50_ < 50 µM) than 1,3,4-oxadiazole-thiazolidienedione hybrids obtained from substituted phenoxy acetic acids hydrazides (**16–21**) against both the tested cell line. Among the synthesised hybrids (**7–15**), compound **9** and **10** bearing halogen at ortho and para position (*o*-Cl, *p*-Br) showed highest cytotoxicity.

**Table 2.** The IC_50_ (µM) of the synthesised compounds (**7–21**) against tested human cancer cell line (MCF-7 and HCT-116)^a^.

Compound	MCF-7^b^	HCT-116^c^
**7**	53.50	57.34
**8**	56.0	56.76
**9**	**7.74**	**12.84**
**10**	**7.87**	**16.14**
**11**	58.37	68.13
**12**	56.28	58.64
**13**	56.3	58.64
**14**	43.37	**42.21**
**15**	47.8	**39.60**
**16**	58.4	67.10
**17**	65.17	55.5
**18**	>100	>100
**19**	61.6	69.50
**20**	>100	>100
**21**	61.95	63.15
**5-FU^d^**	34.82	40.51

^a^IC_50_ values are the concentrations that cause 50% inhibition of cancer cell growth. Data represent the mean values ± standard deviation of three independent experiments performed in triplicate.

^b^Breast cancer (MCF-7); colorectal cancer (HCT-116).

^c^5-FU: 5-florouracil was used as a reference drug (positive control).

Bold values: significant cytotoxicity compared to standard drug 5-FU.

#### *In vitro* thymidylate synthase activity

Compounds (**9**, **10**, **14** and **15**) which showed better cytotoxicity in MTT assay were further tested for their *in vitro* TS activity to confirm its mechanism of action. The TS inhibition results are presented in Table 3. Interestingly, these compounds exhibited remarkable inhibition on the TS enzyme. Compound **9** and **10** revealed TS inhibition with IC_50_ = 1.67 and 2.21 µM, respectively compared to Pemetrexed (IC_50_ = 8.2 µM,). The results of TS inhibition were found to be in agreement with the cytotoxicity results.

**Table 3.** *In vitro* thymidylate synthase (TS) activity of the active compounds **9**, **10**, **14**, **15** and **PTX**.

Compounds	IC_50_ (µM)
**9**	1.67
**10**	2.21
**14**	9.4
**15**	7.9
**PTX**	8.2

IC_50_ values are the mean ± SD of three separate experiments. PTX: Pemetrexed.

#### Molecular docking studies

Molecular docking is an intriguing technique to delineate the mechanism of drug-receptor binding in a time and cost-effective way. Among a number of software’s, AutoDock is known for fast speed, easy availability, and high accuracy (rmsd = 2 Å) results.^32–33^ To support our human TS inhibition results and to get an idea about the binding modes, we carried out shape-based molecular docking studies of compounds **9** and **10** against TS protein (PDB = 6QXG) using Autodock tools. The results of the findings are depicted in Figure 1 and Table 4.

**Table 4.** Docking scores of active compounds **9** and **10** against human thymidylate synthase protein 6QXG.

Compound	Docking score	Amino acid residue
**9**	−9.09	Cys 195, Tyr 135 and Asn 226
**10**	−8.67	Leu 221, Asn 226 and Tyr 230
**5-FU**	−4.22	Phe 80, Arg 78, Thy306 and Ile307

It has been reported that TS-inhibitors act by interacting with the active sites of the receptor through multiple amino acid residues (*viz*. Arg50, Ser216, Asn 226, Asp218, His256, Tyr258, Arg50 and Arg215).^34–35^ Interestingly, our docking results are completely corroborated the literature. We found that the compounds showed good binding affinity towards TS protein (−9.09 and −8.67 kcal/mol for **9** and **10,** respectively) and interacted through multiple H-bonds. Oxygen atom of 4-methoxy group was involved in hydrogen bonding interaction with Cys 195, Tyr 135 and oxadiazole ring oxygen atom with Asn 226 in compound **9**.Whereas in compound **10**, oxygen atom of methoxy group showed hydrogen bonding interaction with Tyr 230, thiazolidinedione C=O group at 4-position showed hydrogen bonding interaction with Asn 226 and nitrogen atom of oxadiazole ring at 4-position with Leu 221. Contrary to this, reference drug 5-fluorouracil (5-FU) exhibited lower affinity (−4.22 kcal/mol) and interacted with Thy 306, Phe 80, Ile307 and Arg 78 *via* H-bonding interaction with C=O group at 2- and 4-position, N–H group at 1- and 3-position respectively. Compounds **9** and **10** have different substituent in the phenyl ring attached to the oxadiazole ring, bearing chlorine at *ortho* position and bromine at *para* position, respectively but due to flexibility of the molecule, compound **10** bend and interact with amino residue Tyr 230, Asn 226 and Leu 221. Whereas, 5 fluorouracil is a small ligand in which all the electronegative atoms interact with the amino acid residues except fluorine. These results were in agreement with the *in vitro* results of compound **9** (IC_50_ = 1.67) and **10** (IC_50_ = 2.21 µM).

## Conclusions

A library of synthesised thiazolidinedione-1,3,4-oxadiazole hybrids have been screened for *in vitro* antiproliferative as well as TS activities. All the synthesised hybrids follow Lipinski and Veber rules indicating good drug likeness properties upon oral administration. Among the synthesised hybrids, compound **9** and **10** showed excellent cytotoxicity against MCF-7 and HCT-116 cells. From the *in vitro* TS activity, Compound **9** and **10** were found to be potent inhibitors against TS protein with IC_50_ = 1.67 and 2.21 µM, respectively. The docking studies of compounds **9** and **10** were found to be consistent with *in vitro* cytotoxicity and TS results. From these studies, it can be concluded that compounds **9** and **10** have the potential to be developed as TS inhibitors.

## Supplementary Material

Supplemental Material
