# Supplementary material for: Design, synthesis and in vitro antiproliferative activity of new thiazolidinedione-1,3,4-oxadiazole hybrids as thymidylate synthase inhibitors
Source: J Enzyme Inhib Med Chem. 2020 Apr 30;35(1):1116–23. doi: 10.1080/14756366.2020.1759581 (PMC7241536; doi:10.1080/14756366.2020.1759581)
Supplement: Supplemental Material [file IENZ_A_1759581_SM9042.pdf]

SUPPLEMENTARY MATERIAL

Design, Synthesis and *in vitro* antiproliferative activity of new thiazolidinedione-1, 3, 4-oxadiazole hybrids as thymidylate synthase inhibitors

Zohor Mohammad Mahdi Alzhrani[a], Mohammad Mahboob Alam[a], Thikryat Neamatallah[b], Syed Nazreen[a]

Procedure for synthesis of (E)-5-(4-methoxybenzylidene)thiazolidine-2,4-dione (4)

A mixture of thiazolidinedione **3** (0.05 mole, 5.85 g) and anisaldehyde (0.05 mole, 6.0 ml) in ethanol (50 ml) was taken and cold NaOH solution (40%, 10 ml) was added drop wise into it. The reaction was kept overnight for 15 h. The resulting solid was acidified with hydrochloric acid, filtered, washed with excess water and dried. It was recrystallized with ethanol to give yellow crystals. Yield 90% mp 217-218 ºC; ^{1}H NMR (300 MHz, DMSO-d_6 , δ ppm): 3.82 (s, 3H), 7.09 (d, 2H, J = 8.7 Hz), 7.56 (d, 2H, J = 8.7 Hz), 7.75 (s, 1H), 11.92 (s,1H). ^{13}C NMR (75 MHz, DMSO-d_6 , δ ppm): 55.95, 115.38, 120.75, 125.95, 132.30, 132.53, 161.45, 167.88, 168.40. ESI -ve MS (m/z): 234 $(M-H)^{+}$.

Procedure for the synthesis of ethyl 2-((E)-5-(4-methoxybenzylidene)-2,4-dioxothiazolidin-3-yl)acetate (5).

A mixture of compound **4** (0.05 mole), ethyl chloroacetate (0.05 mole) and anhydrous potassium carbonate (0.075 mole) in dry acetone (100 ml) was stirred and refluxed for 15 h. After reaction completion, the content was filtered under hot condition. The filtrate so obtained was

concentrated to give compound **5** as yellow solid. Yield: 75% mp 132-133ºC. ^{1}H NMR (300 MHz, DMSO-d_6 , δ ppm): 1.21 (t, 3H, *J* = 6.9 Hz), 3.84 (s, 3H), 4.17 (q, 2H, *J* = 7.2, 14.1 Hz), 4.47 (s, 2H), 7.12 (d, 2H, *J* = 8.7 Hz), 7.60 (d, 2H, *J* = 8.7 Hz), 7.95 (s, 1H). ^{13}C NMR (75 MHz, DMSO-d_6 , δ ppm): 14.38, 42.60, 56.00, 62.15, 115.51, 117.61, 125.61, 132.94, 134.59, 161.88, 165.52, 167.24, 167.47. ESI +ve MS (m/z): 322 $(M+H)^+$.

Procedure for synthesis of 2-((E)-5-(4-methoxybenzylidene)-2,4-dioxothiazolidin-3-yl)acetic acid (6).

A mixture of compound **5** (0.05 mole), glacial acetic acid (20 ml) and 11 N HCl (20 ml) was stirred at 90-100 ^{0}C for 8 h. After complete hydrolysis, the mixture was concentrated to afford yellow solid which was filtered, washed with cold water and dried. It was recrystallized with methanol to give pure acid derivative **6**. Yield 80% mp 221-222ºC. ^{1}H NMR (300 MHz, DMSO-d_6 , δ ppm): 3.86 (s, 3H), 4.37 (s, 2H), 7.13 (d, 2H, *J* = 8.4 Hz), 7.63 (d, 2H, *J* = 8.7 Hz), 7.95 (s, 1H), 13.44 (s, 1H). ^{13}C NMR (75 MHz, DMSO-d_6 , δ ppm): 37.34, 50.66, 110.15, 112.47, 120.35, 127.54, 129.02, 156.49, 160.26, 162.13, 163.15. ESI -ve MS (m/z):292 $(M-H)^+$.

IR data of the target compounds 7-21:

(Z)-5-(4-methoxybenzylidene)-3-((5-phenyl-1,3,4-oxadiazol-2-yl)methyl)thiazoli dine-2,4-dione (7). IR (ν_{max}, cm^{-1}): 3016, 2977, 2933, 2571, 1731, 1709, 1679, 1588, 1441, 1309, 1256, 1174, 1149, 1079, 860, 525;

(Z)-5-(4-methoxybenzylidene)-3-((5-(3-chlorophenyl)-1,3,4-oxadiazol-2-yl)methyl)thiazoli dine-2,4-dione (8). IR (ν_{max}, cm^{-1}): 3014, 2932, 1732, 1682, 1587, 1568, 1509, 1456, 1421, 1373, 1308, 1256, 1175, 1143, 1094, 1024, 832;

(Z)-5-(4-methoxybenzylidene)-3-((5-(2-chlorophenyl)-1,3,4-oxadiazol-2-yl)methyl)thiazolidine-2,4-dione (9). IR (ν_{max}, cm^{-1}): 3024, 1737, 1684, 1607, 1590, 1512, 1490, 1425, 1401, 1380, 1263, 1152, 1180, 1097, 824;

(Z)-5-(4-methoxybenzylidene)-3-((5-(4-bromophenyl)-1,3,4-oxadiazol-2-yl)methyl)thiazolidine-2,4-dione (10). IR (ν_{max}, cm^{-1}): 3025, 2977, 2933, 1739, 1684, 1604, 1588, 1566, 1511, 1406, 1382, 1323, 1260, 1177, 1151, 1097, 829;

(Z)-5-(4-methoxybenzylidene)-3-((5-p-tolyl-1,3,4-oxadiazol-2-yl)methyl)thiazolidine-2,4-dione (11). IR (ν_{max}, cm^{-1}): 3050, 2845, 1749, 1685, 1609, 1593, 1509, 1500, 1422, 1369, 1301, 1259, 1174, 1140, 1093, 1010, 830;

(Z)-5-(4-methoxybenzylidene)-3-((5-(2-hydroxyphenyl)-1,3,4-oxadiazol-2-yl)methyl)thiazolidine-2,4- dione (12). IR (ν_{max}, cm^{-1}): 3263, 2950, 1749, 1686, 1585, 1549, 1511, 1489, 1411, 1261, 1174, 1160, 1086, 1070, 831;

(Z)-5-(4-methoxybenzylidene)-3-((5-o-tolyl-1,3,4-oxadiazol-2-yl)methyl)thiazolidine-2,4-dione (13). IR (ν_{max}, cm^{-1}): 3014, 2950, 1723, 1674, 1589, 1543, 1512, 1425, 1417, 1395, 1368, 1334, 1311, 1260, 1176, 1135, 1089, 1020, 822;

(Z)-5-(4-methoxybenzylidene)-3-((5-(3-nitrophenyl)-1,3,4-oxadiazol-2-yl)methyl)thiazolidine-2,4-dione (14). IR (ν_{max}, cm^{-1}): 3087, 2838, 1733, 1679, 1587, 1527, 1509, 1374, 1348, 1305, 1258, 1178, 1141, 1091, 1067, 1022, 833;

(Z)-5-(4-methoxybenzylidene)-3-((5-m-tolyl-1,3,4-oxadiazol-2-yl)methyl)thiazolidine-2,4-dione (15). IR (ν_{max}, cm^{-1}): 2931, 1697, 1683, 1606, 1585, 1511, 1370, 1338, 1253, 1148, 1025, 829;

(Z)-5-(4-methoxybenzylidene)-3-((5-(phenoxymethyl)-1,3,4-oxadiazol-2-yl)methyl)thiazolidine-2,4-dione (16). IR (ν_{max}, cm^{-1}): 3030, 2937, 1734, 1685, 1587, 1512, 1490, 1373, 1305, 1261, 1176, 1144, 1034, 829;

(Z)-3-((5-((3-chlorophenoxy)methyl)-1,3,4-oxadiazol-2-yl)methyl)-5-(4-methoxybenzylidene)thiazolidine-2,4-dione (17). IR (ν_{max}, cm^{-1}): 3017, 2935, 1732, 1683, 1589, 1511, 1482, 1456, 1373, 1338, 1257, 1209, 1175, 1146, 1097, 1023, 825;

(Z)-3-((5-((2,3-dichlorophenoxy)methyl)-1,3,4-oxadiazol-2-yl)methyl)-5-(4-methoxybenzylidene)thiazolidine-2,4-dione (18). IR (ν_{max}, cm^{-1}): 3020, 2928, 1732, 1687, 1583, 1517, 1487, 1373, 1305, 1260, 1174, 1138, 1014, 825;

(Z)-5-(4-methoxybenzylidene)-3-((5-((naphthalen-1-yloxy)methyl)-1,3,4-oxadiazol-2-yl)methyl) thiazolidine-2,4-dione (19). IR (ν_{max}, cm^{-1}): 3027, 2977, 2933, 1731, 1682, 1589, 1510, 1421, 1382, 1402, 1257, 1174, 1150, 1098, 826;

(Z)-5-(4-methoxybenzylidene)-3-((5-((naphthalen-3-yloxy)methyl)-1,3,4-oxadiazol-2-yl)methyl) thiazolidine-2,4-dione (20). IR (ν_{max}, cm^{-1}): 3015, 2978, 2933, 1731, 1711, 1679, 1589, 1567, 1510, 1421, 1402, 1382, 1256, 1174, 1124, 1150, 1098, 1058, 825;

(Z)-5-(4-methoxybenzylidene)-3-((5-((quinolin-8-yloxy)methyl)-1,3,4-oxadiazol-2-yl)methyl) thiazolidine-2,4-dione (21). IR (ν_{max}, cm^{-1}): 3025, 2977, 1736, 1683, 1588, 1569, 1511, 1497, 1471, 1422, 1369, 1336, 1309, 1266, 1211, 1141, 1107, 1084, 1026, 1001, 817;

Antiproliferative activity

Cell Lines and Culture Medium

Two human cancer cell lines, MCF-7 and HCT-116 were cultured in Dulbecco's Modified Eagles Medium (DMEM) high glucose medium which was supplemented with 10% fetal bovine serum (FBS), 10,000 units/mL penicillin/streptomycin (Pen/Strep) and 1% glutamine). The above cell lines were cultured in 75 cm^2 flasks and maintained at 37 °C in an incubator humidified with 5% CO_2. The cell culture was performed using aseptic techniques in a Class II Safety Flow Hood.

Cytotoxicity assay

Breast MCF-7 and colorectal HCT-116 cancer cells were added at (1×10^5 cells/mL) into a 96 well plate with three replicates and incubated overnight for attachment at 37°C, in 5% CO_2 humidified atmosphere. Drug concentrations at 6 serial dilutions (100, 50, 10, 1, 0.5 and 0.1 μM) were added in triplicates and incubated at 37°C, 5% CO_2 for 72 h. Drugs were dissolved in 0.1 % DMSO as a vehicle. Untreated cells were used as control. 5-fluorouracil (5-FU) was used as a positive control. Thereafter, each well for each time point was removed and replaced with 100 μM of full medium containing 10% of 3-(4,5- dimethylthiaxolyl-2)-2,5-diphenyltetrazoliumbromide (MTT) (10 mg/mL). Media was removed and 100μl of DMSO was added and cells were incubated for further 5 mins at 37 ^{o}C and 5% CO_2. Plates were quantified using the SpectraMax M3 plate reader at 570 nm. The percentage inhibition was calculated as 100–[(Mean OD of treated cell × 100)/Mean OD of vehicle treated cells (DMSO)]. All the experiments were repeated at least three independent experiments. The results are presented in Table 2.

***In vitro* Thymidylate synthase assay**

It involves a mixture containing 2-mercaptoethanol (0.1 M), (6R,S)-tetrahydrofolate (0.0003 M), formaldehyde (0.012 M), $MgCl_2$ (0.02 M), dUMP (0.001 M), TrisHCl (0.04 M), and NaEDTA

(0.00075 M). This assay was done spectrophotometrically at 30° C and pH 7.4. The reaction was initiated by the addition of an amount of enzyme giving a change in absorbance at 340 nm of 0.016/min in the absence of inhibitor. The percent inhibition was determined at a minimum of four inhibitor concentrations within 20% of the 50% point. The standard deviations for determination of the 50% points were within ±10% of the values given.

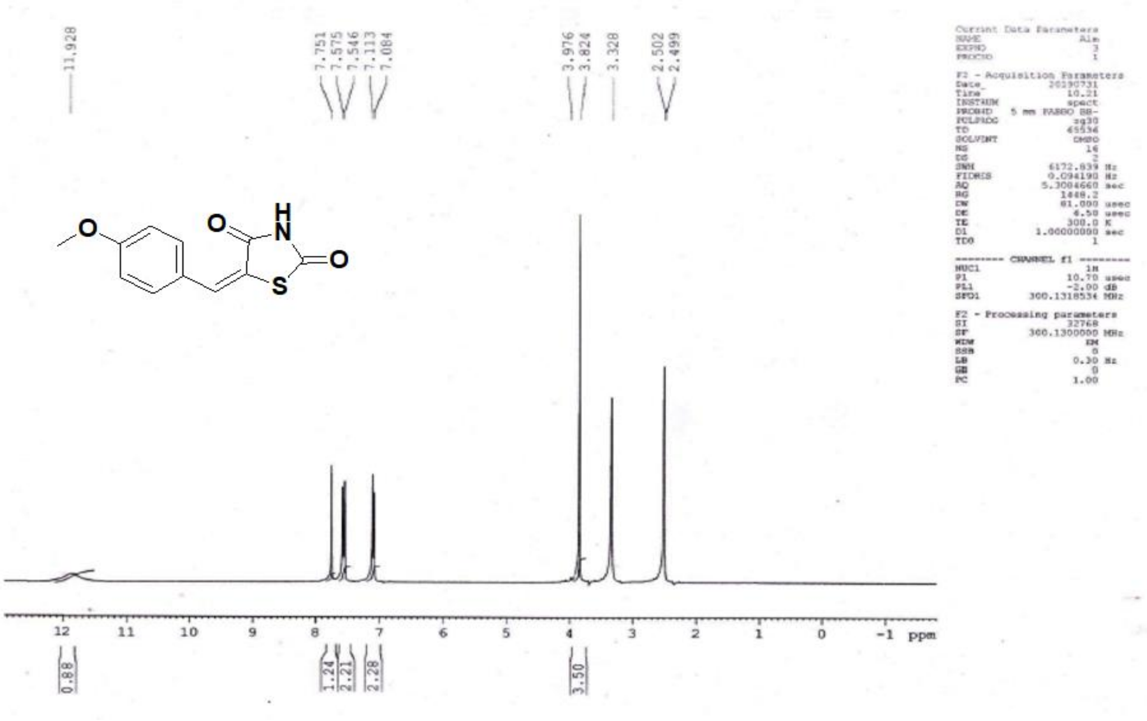

^{1}H NMR of compound 4

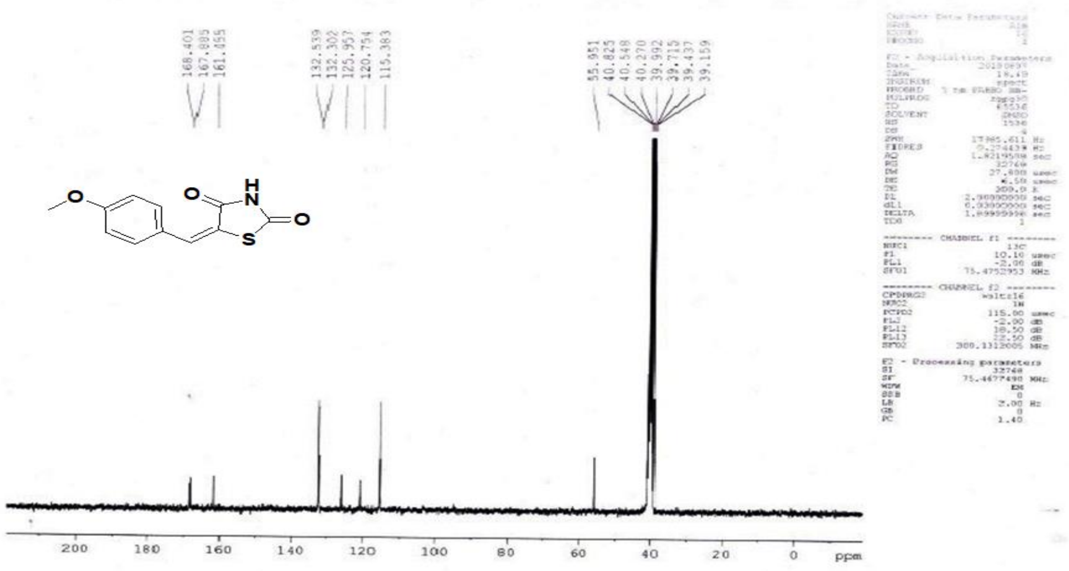

^{13}C NMR of compound 4

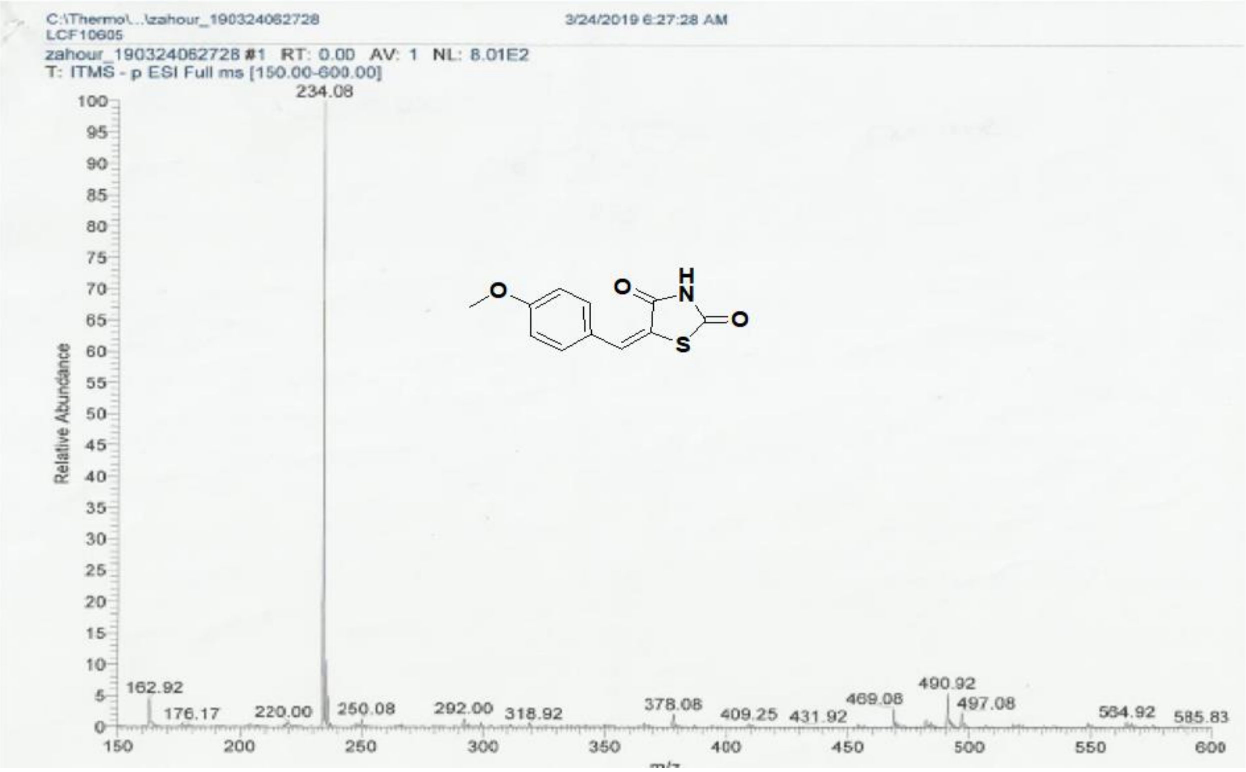

Mass of compound 4

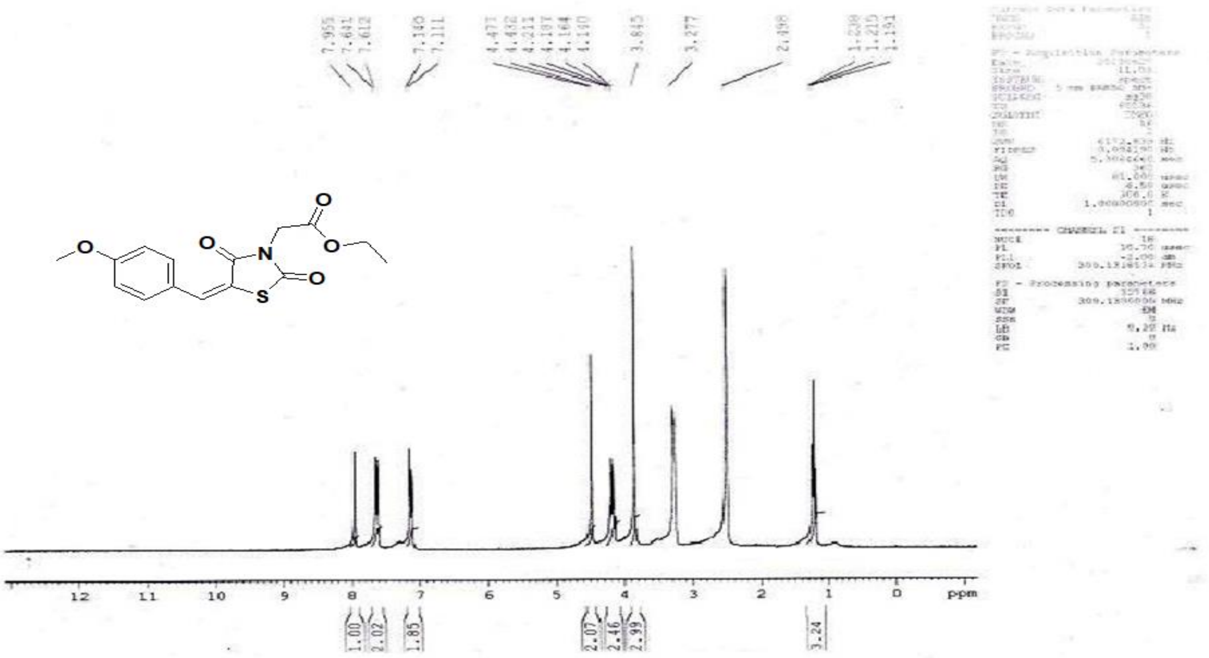

^{1}H NMR of compound 5

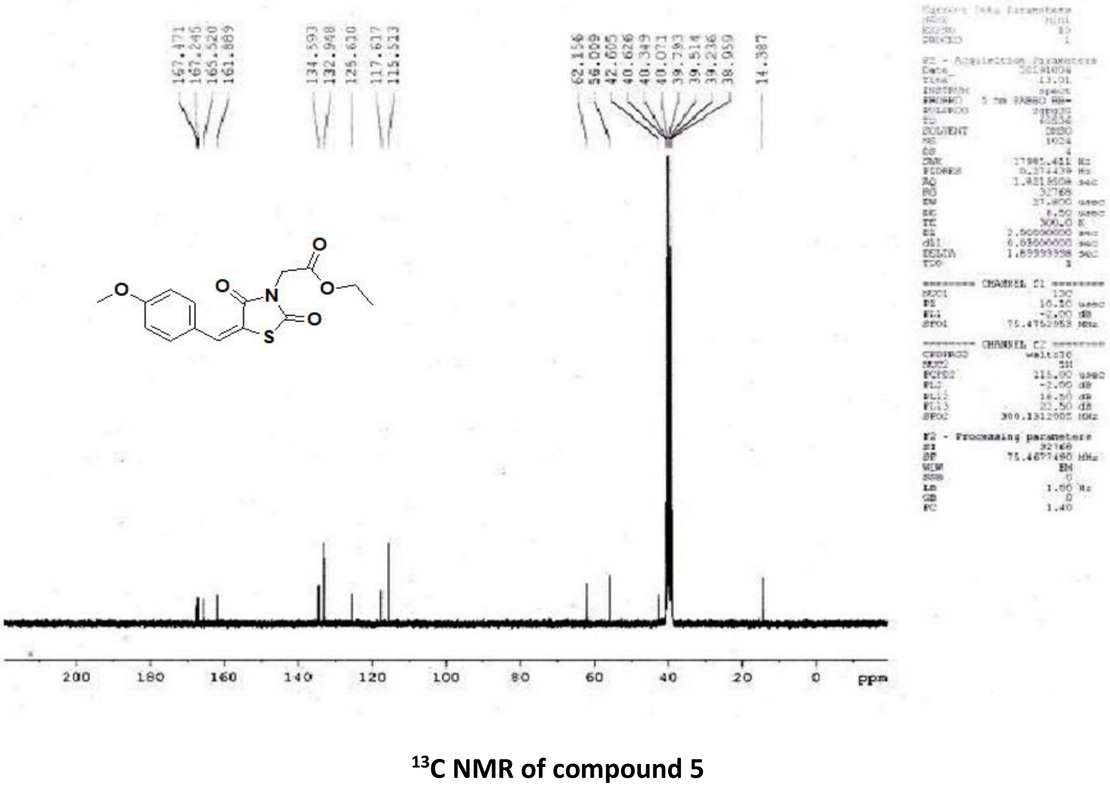

^{13}C NMR of compound 5

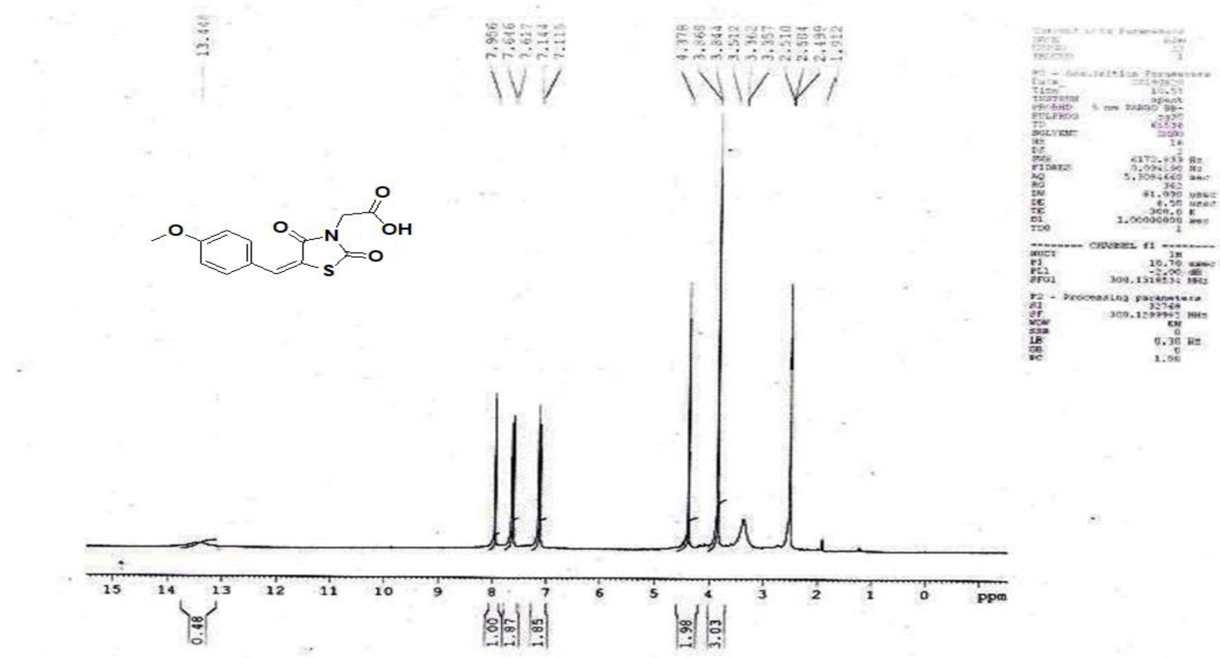

^{1}H NMR of compound 6

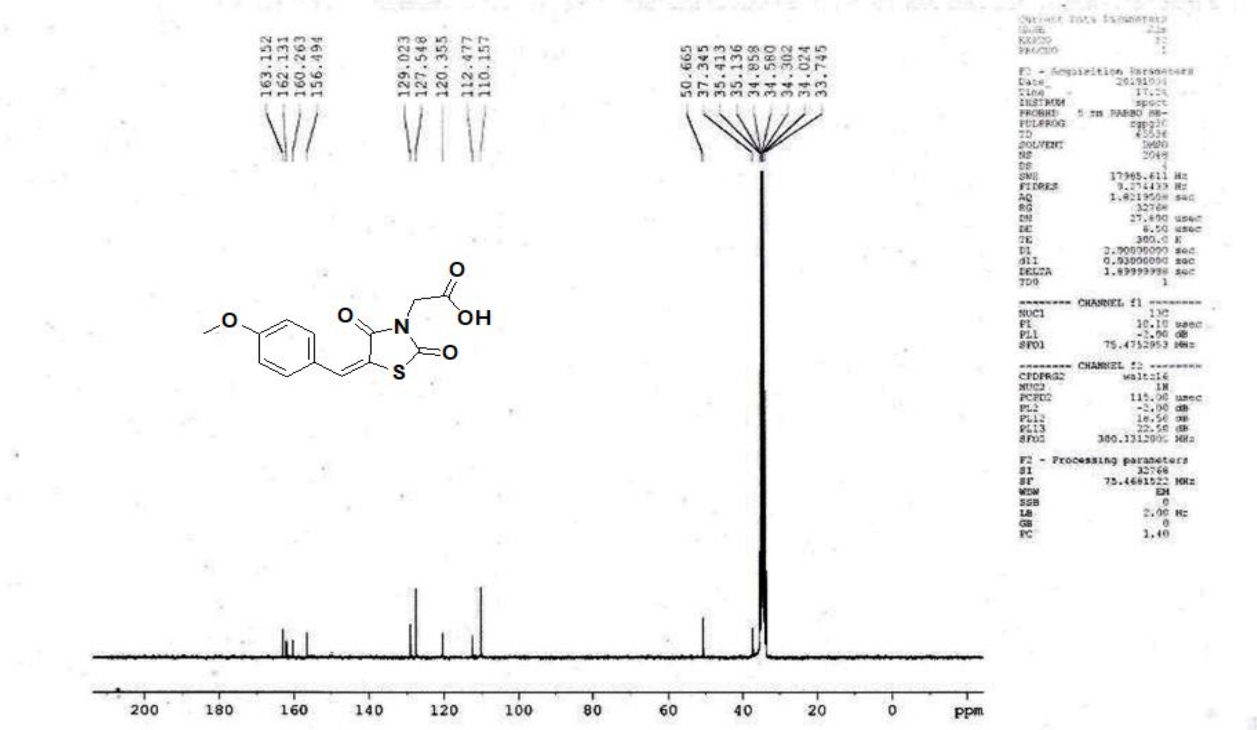

^{13}C NMR of compound 6

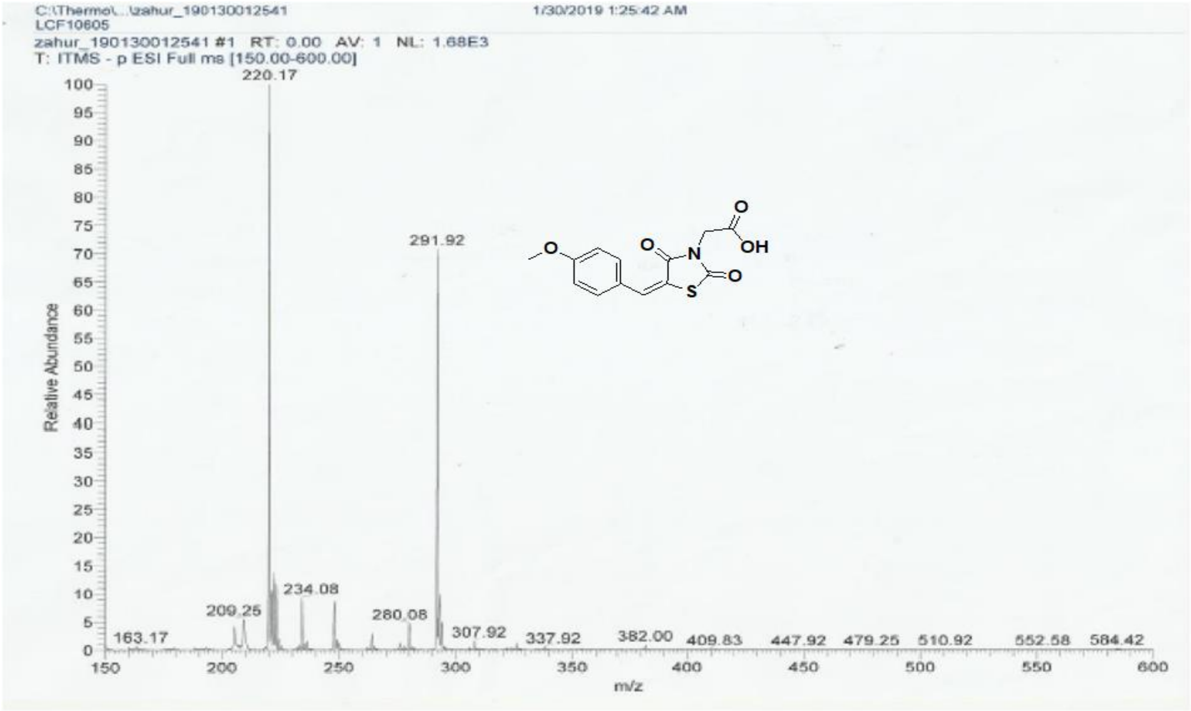

Mass of compound 6

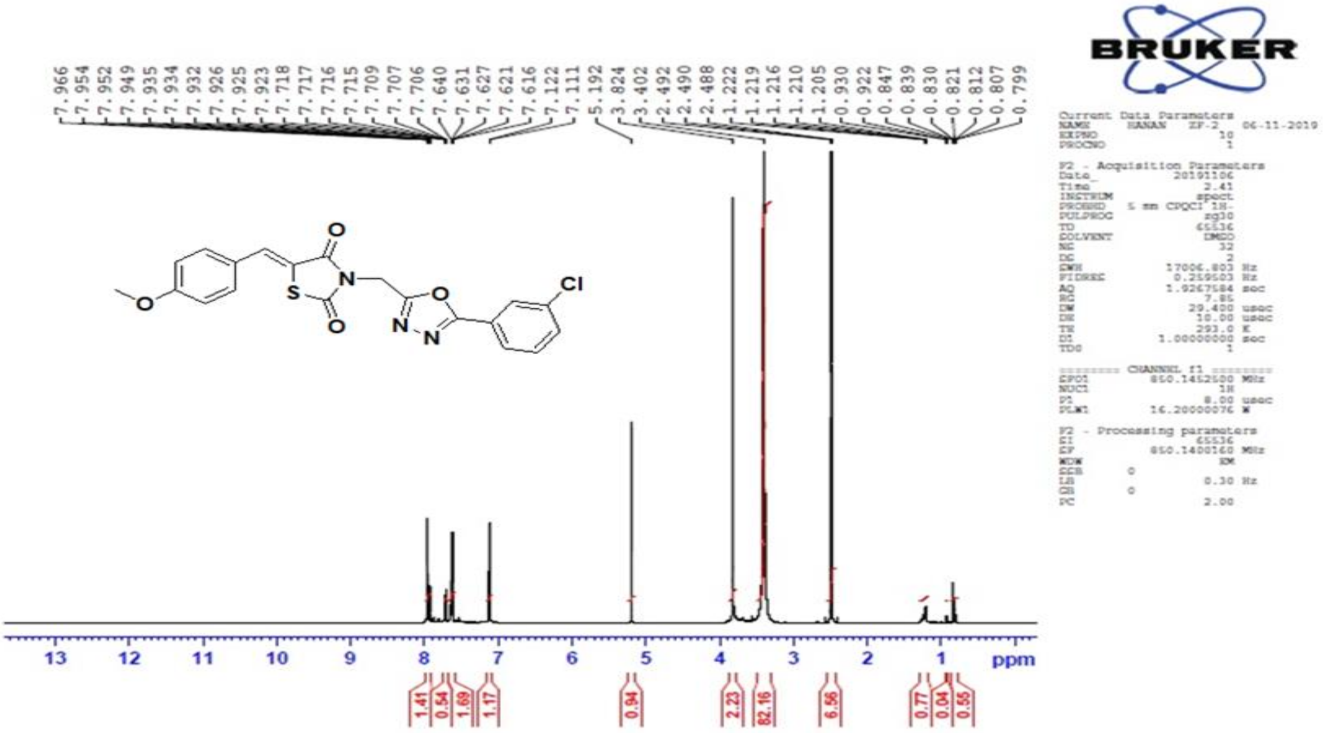

^{1}H NMR of compound 8

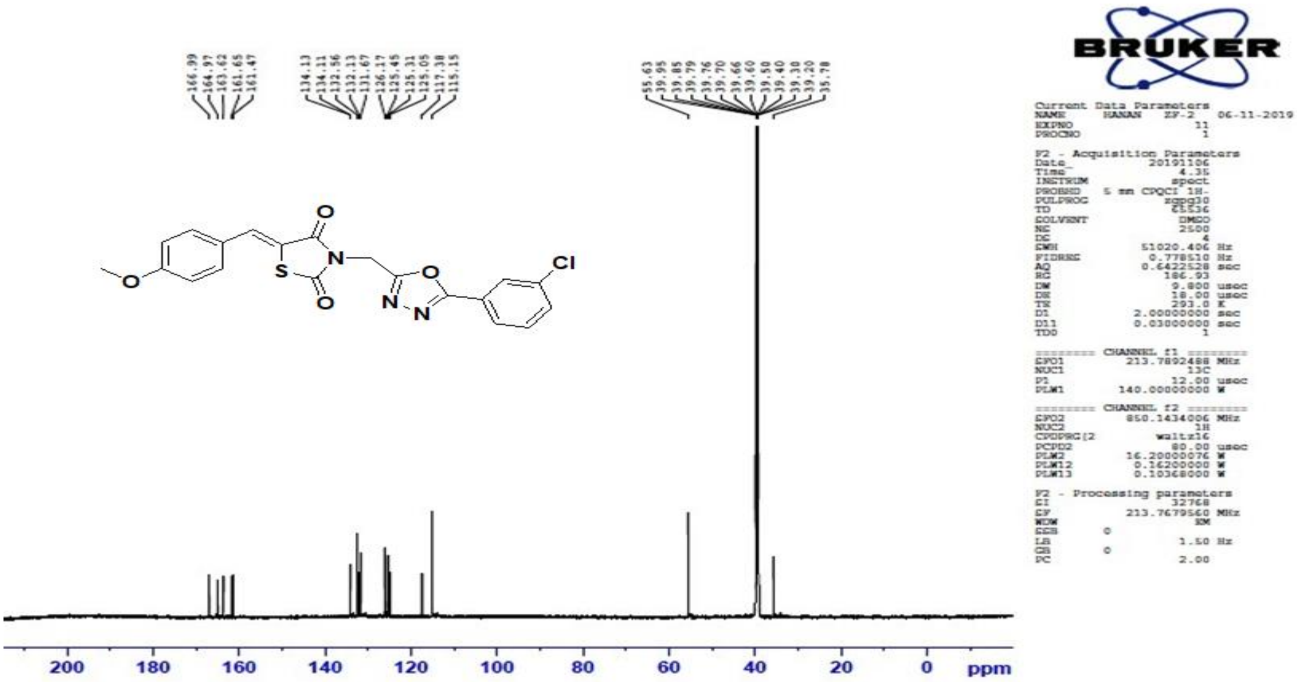

^{13}C NMR of compound 8

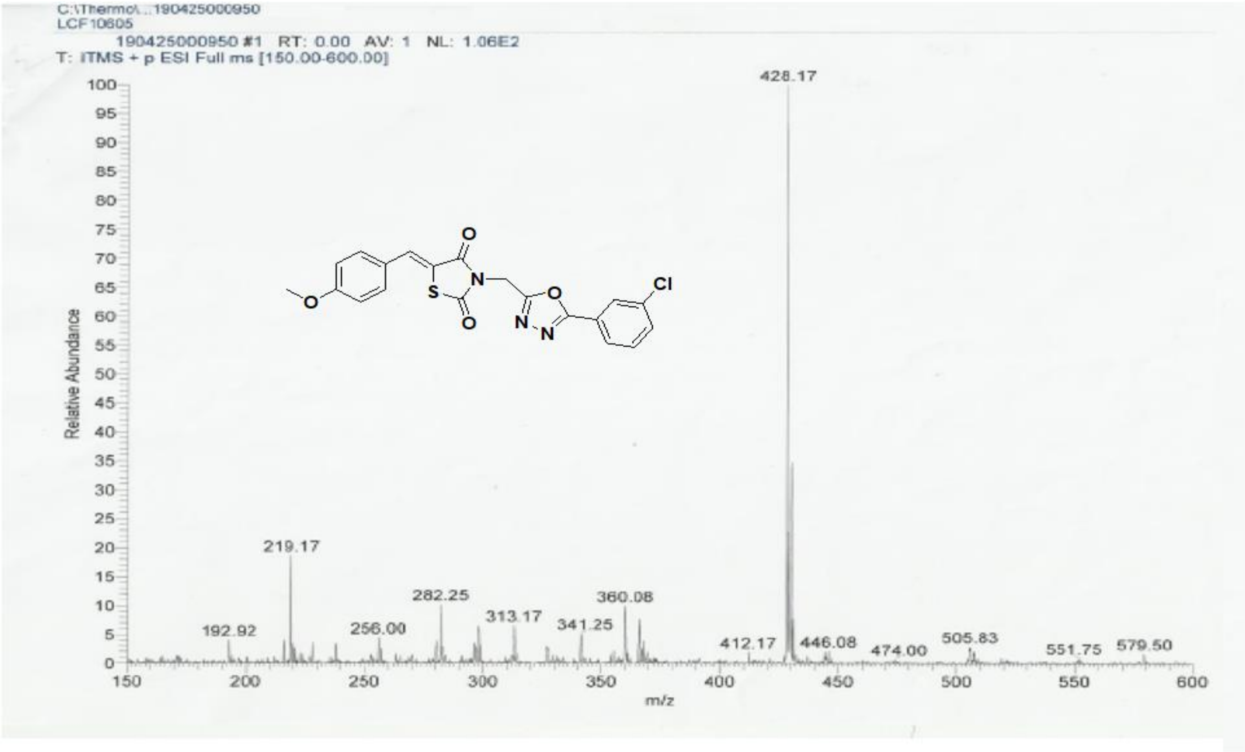

Mass of compound 8

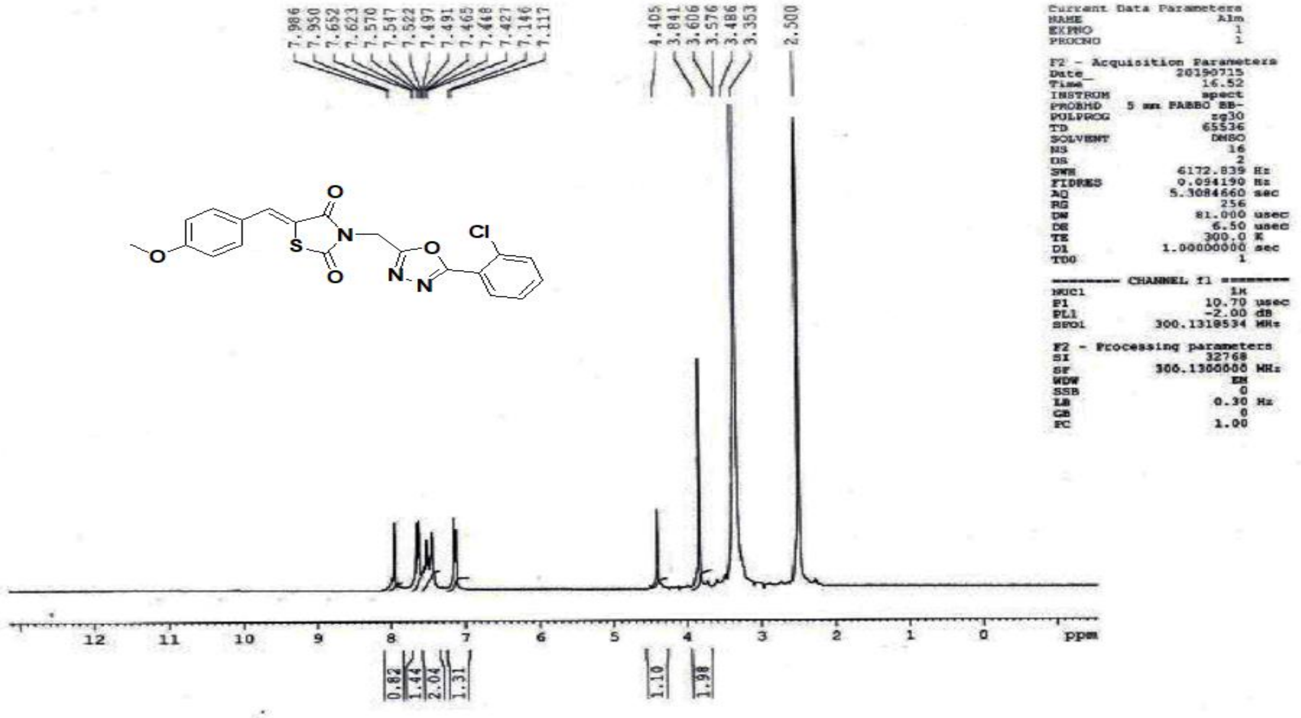

^{1}H NMR of compound 9

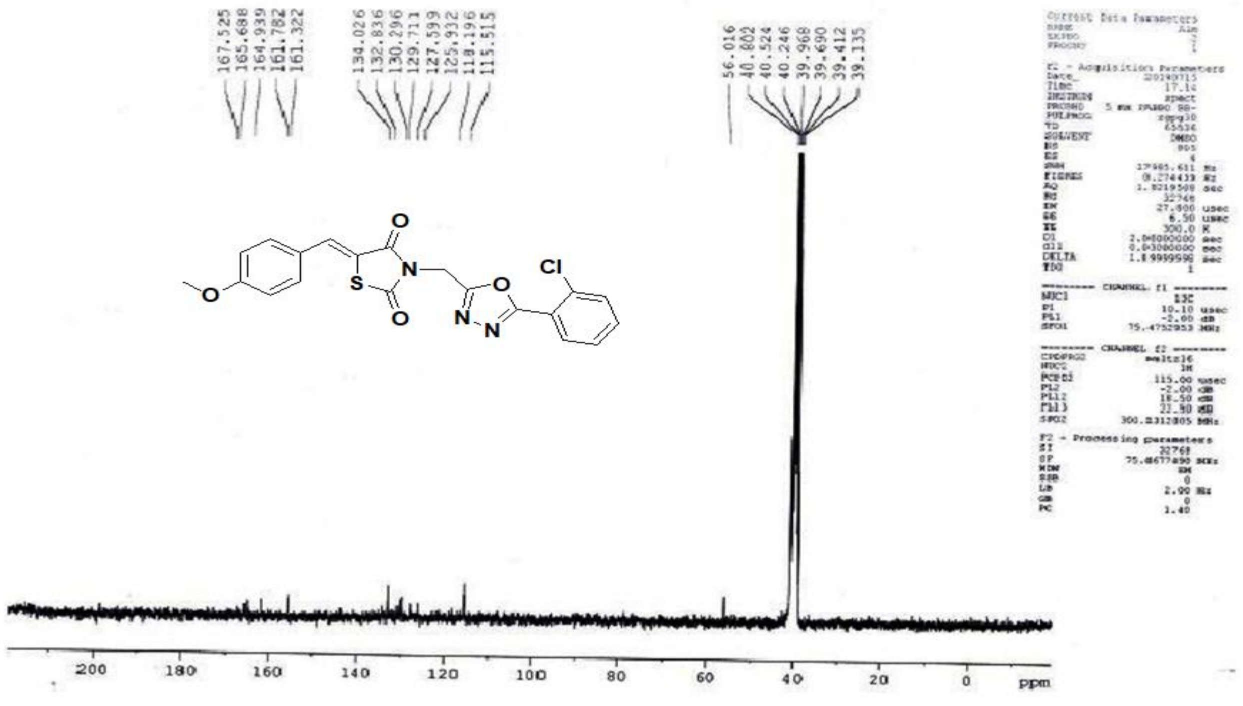

^{13}C NMR of compound 9

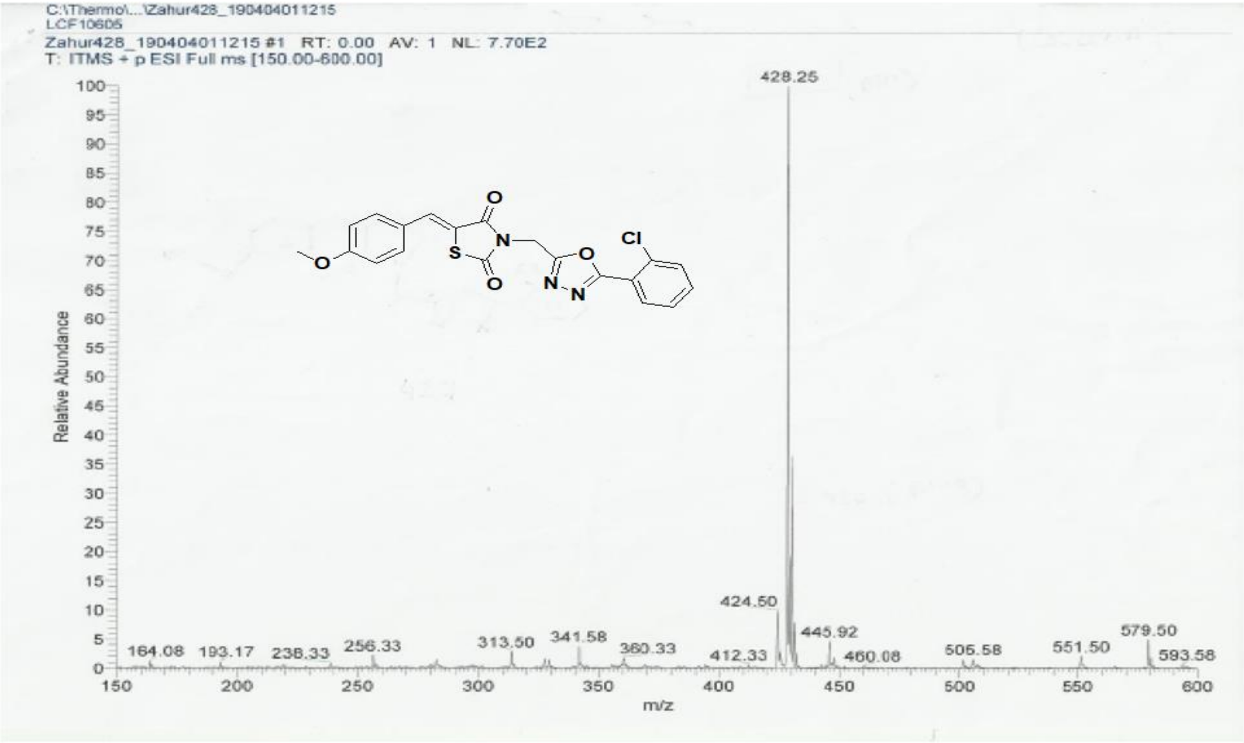

Mass of compound 9

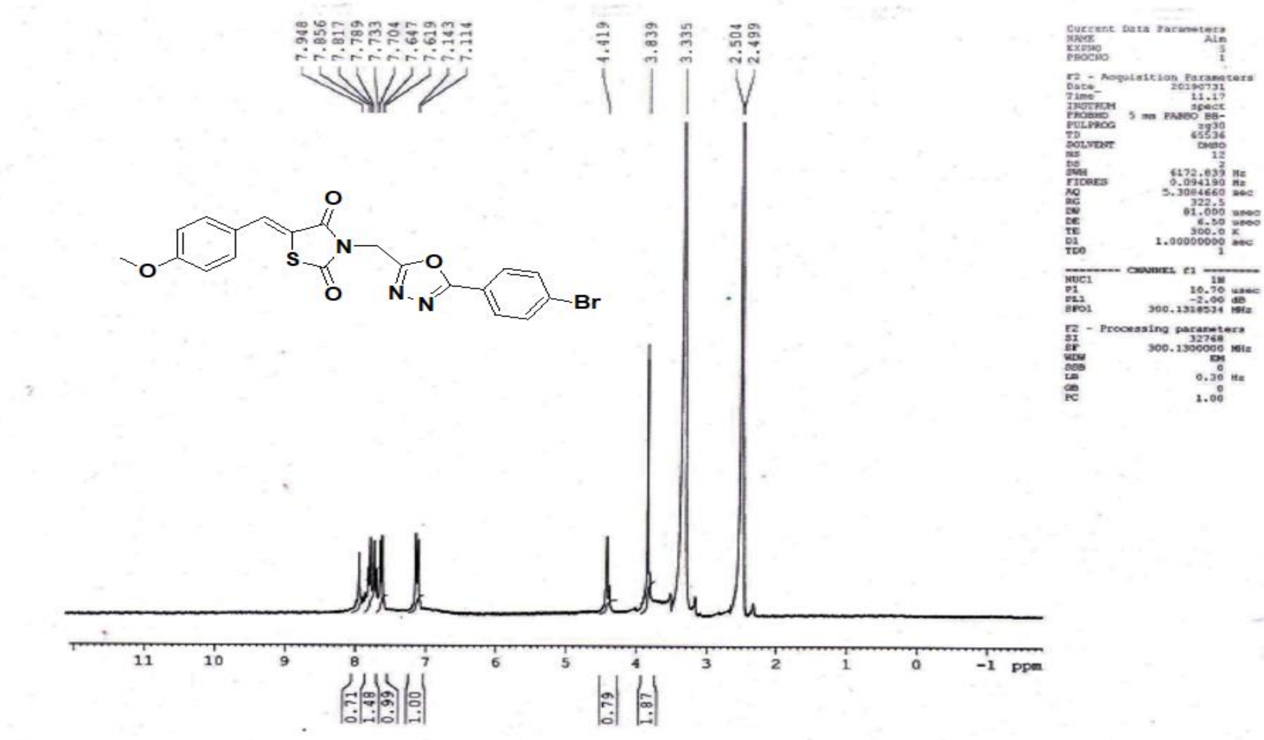

^{1}H NMR of compound 10

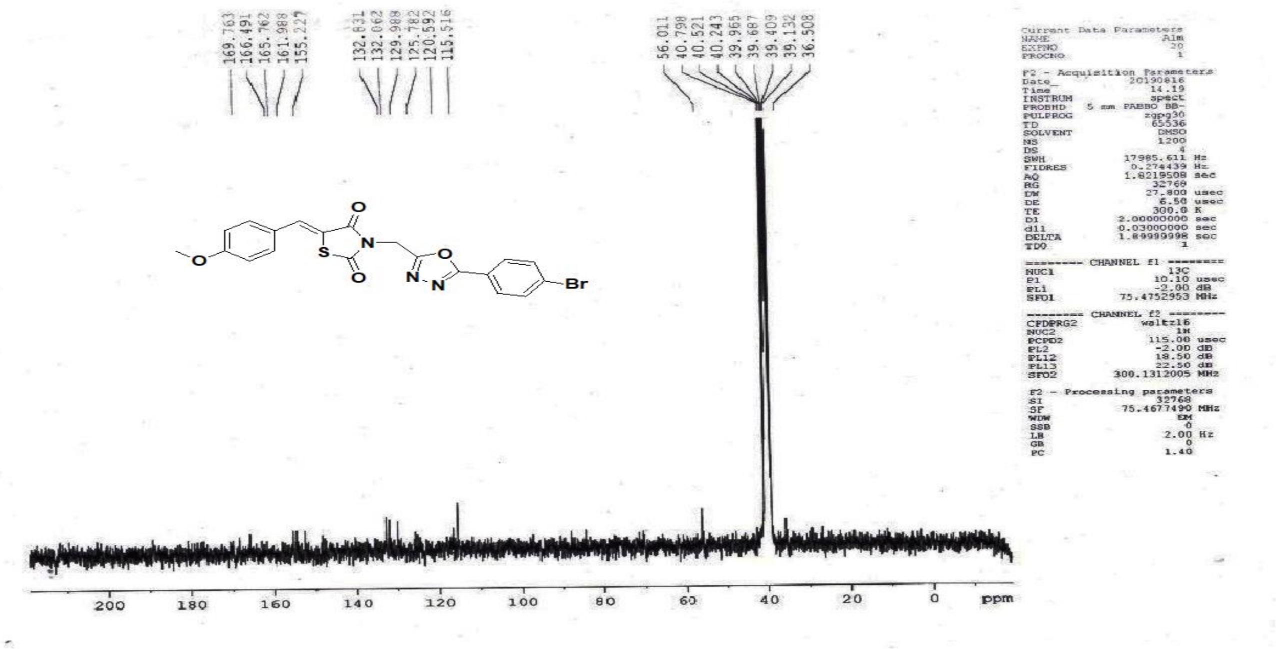

^{13}C NMR of compound 10

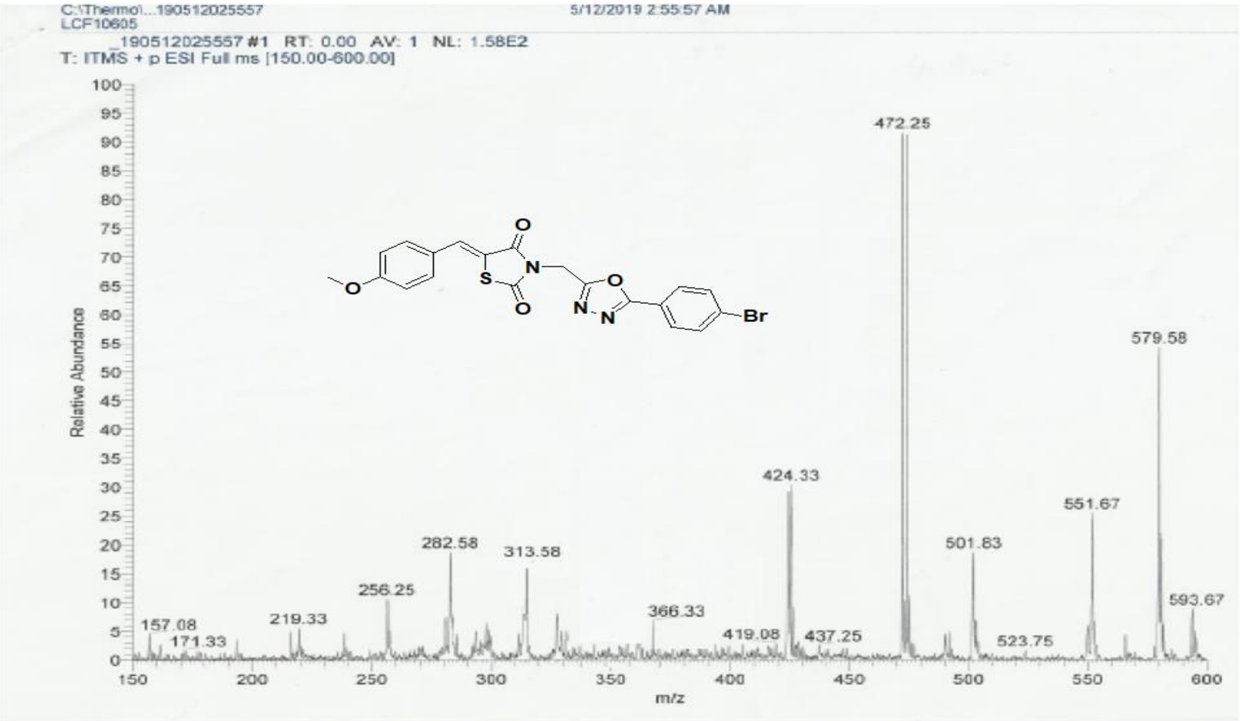

Mass of compound 10

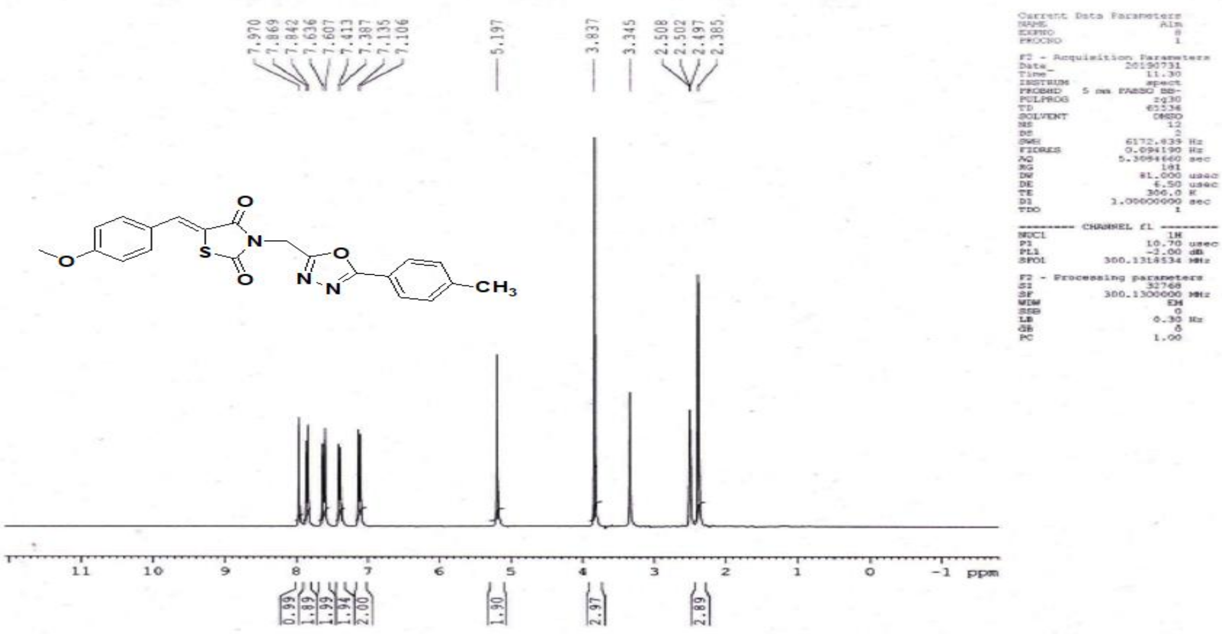

^{1}H NMR of compound 11

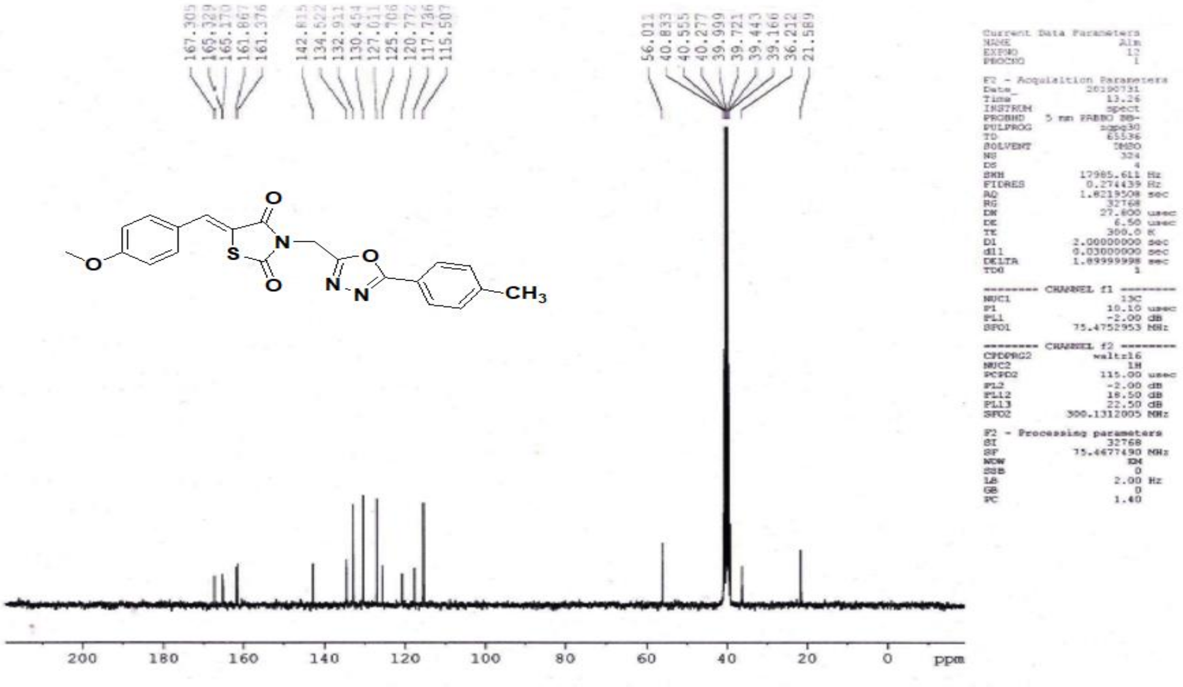

^{13}C NMR of compound 11

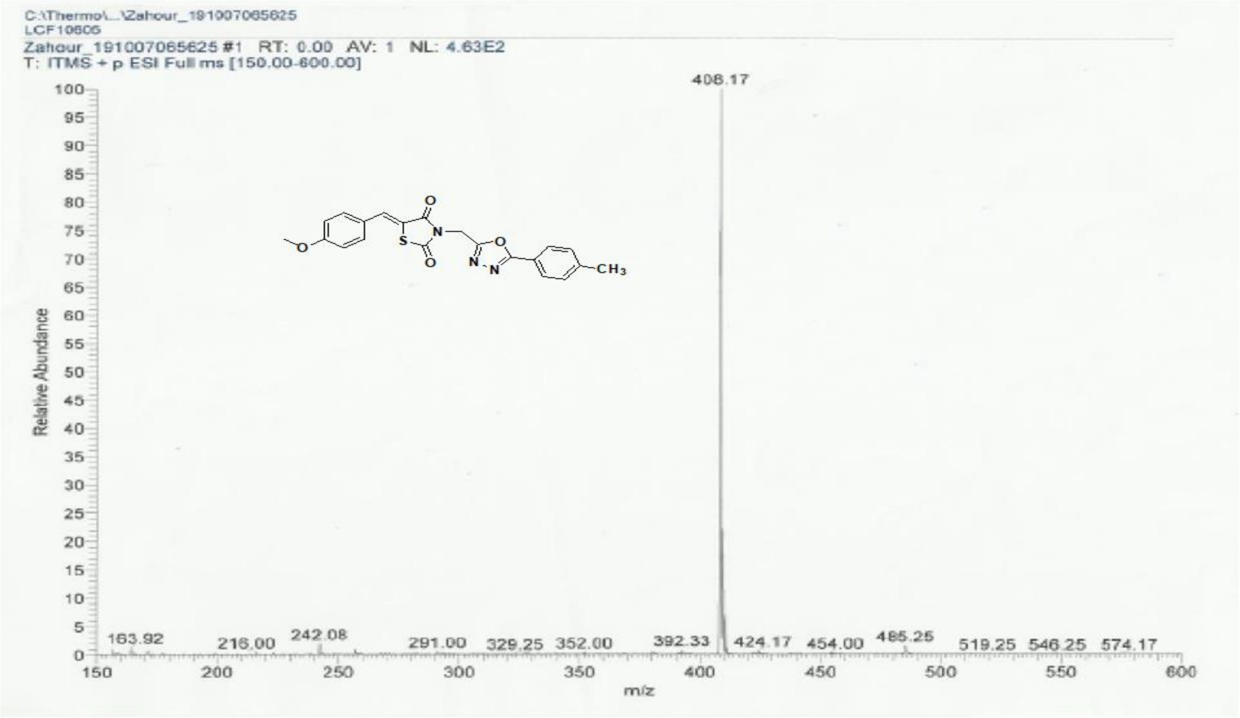

Mass of compound 11

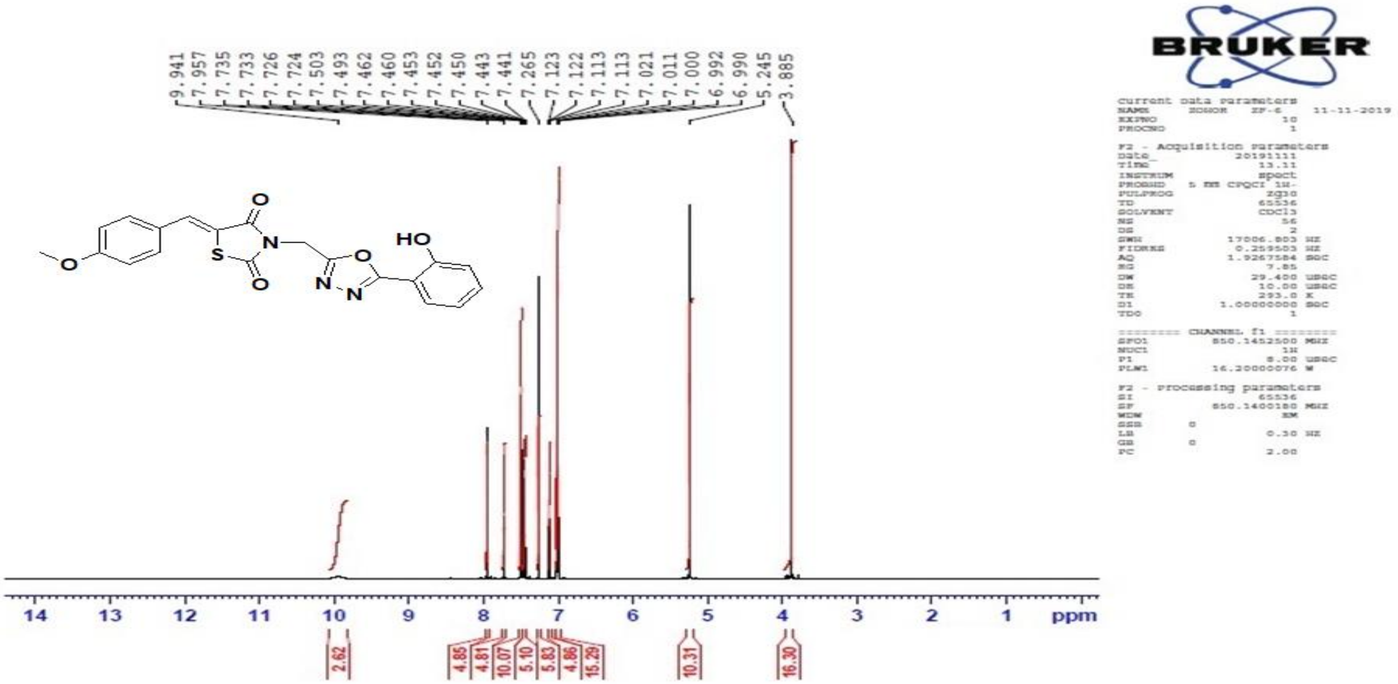

^{1}H NMR of compound 12

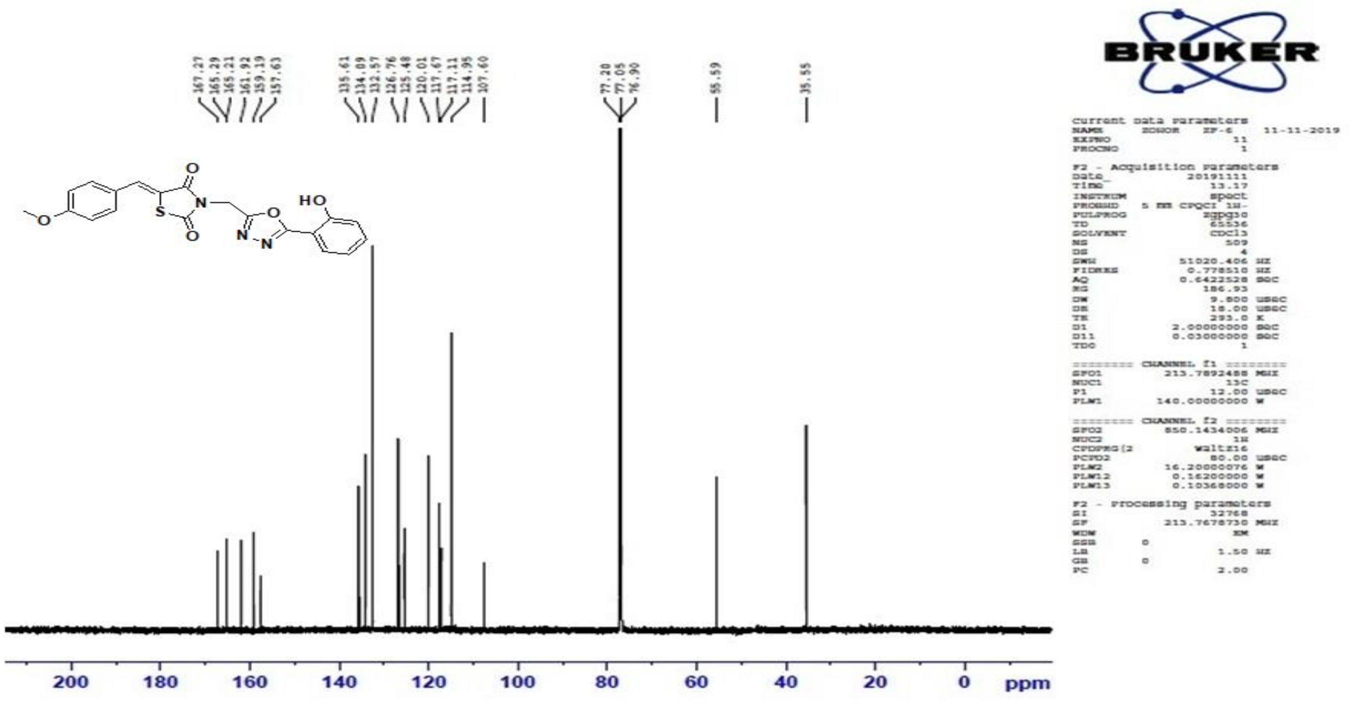

^{13}C NMR of compound 12

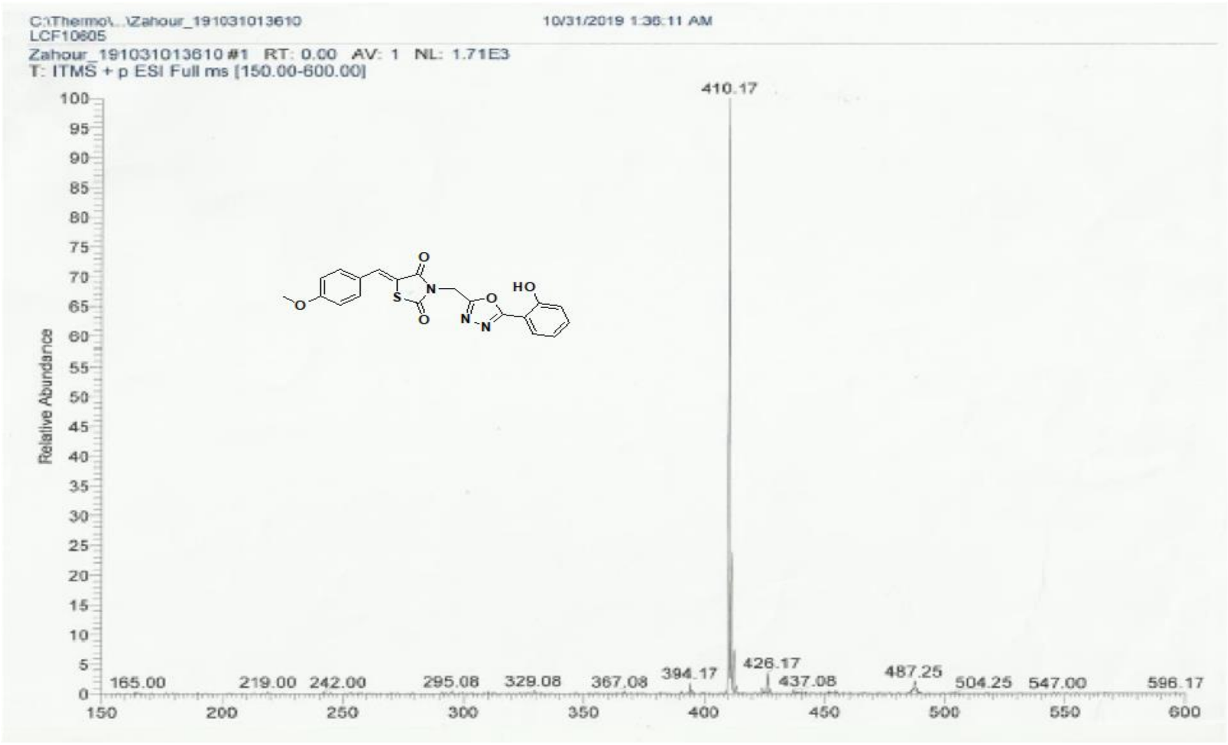

Mass of compound 12

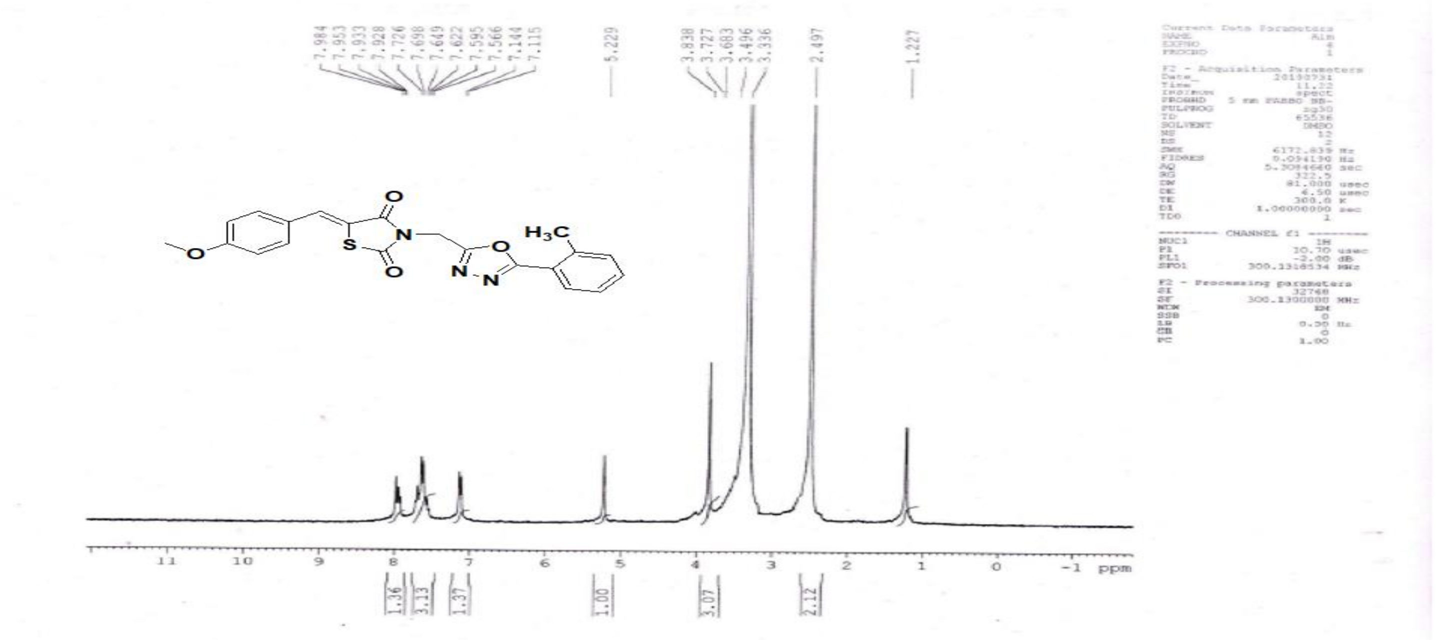

^{1}H NMR of compound 13

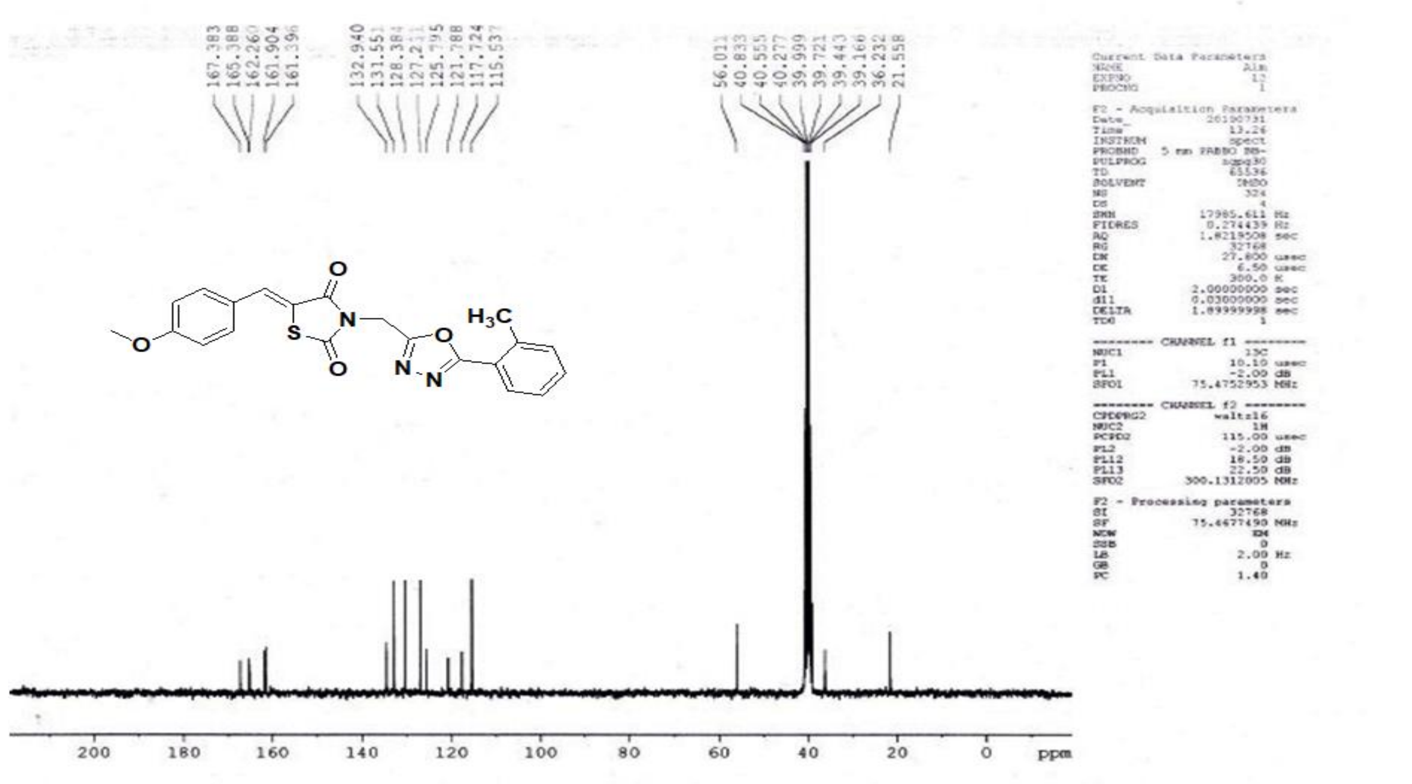

^{13}C NMR of compound 13

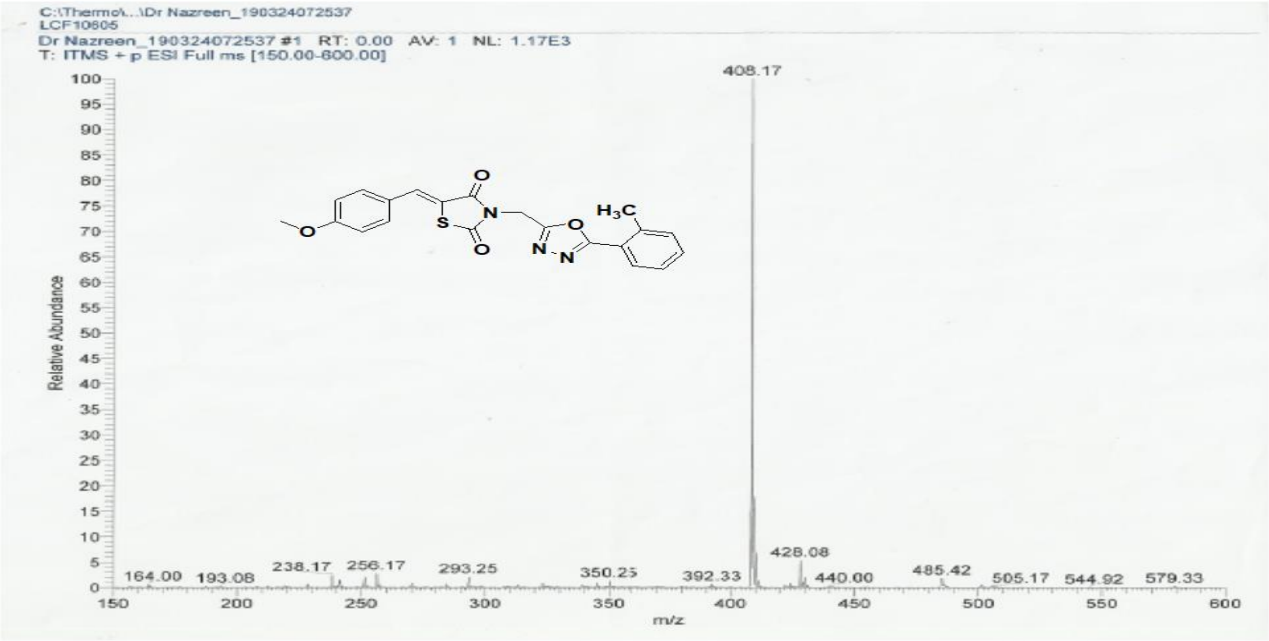

Mass of compound 13

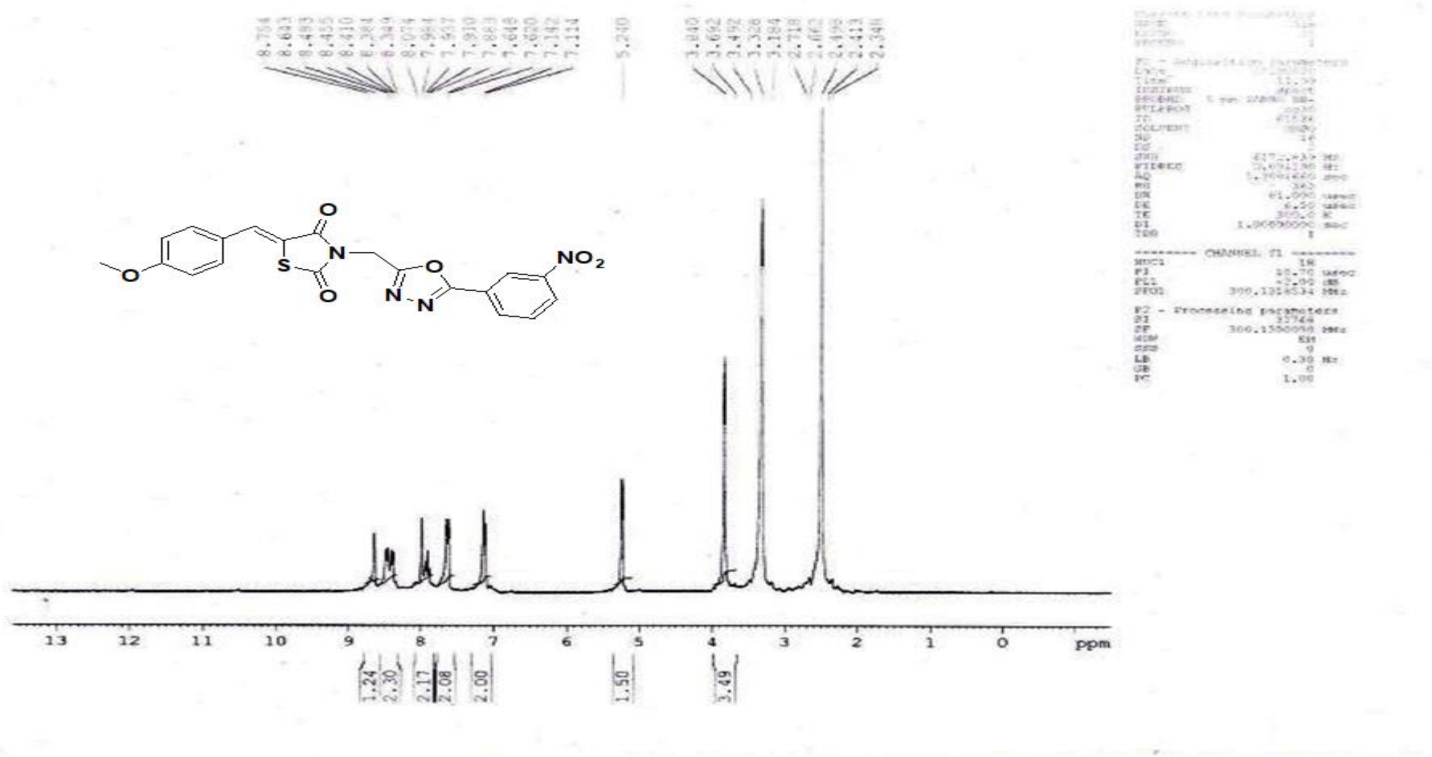

^{1}H NMR of compound 14

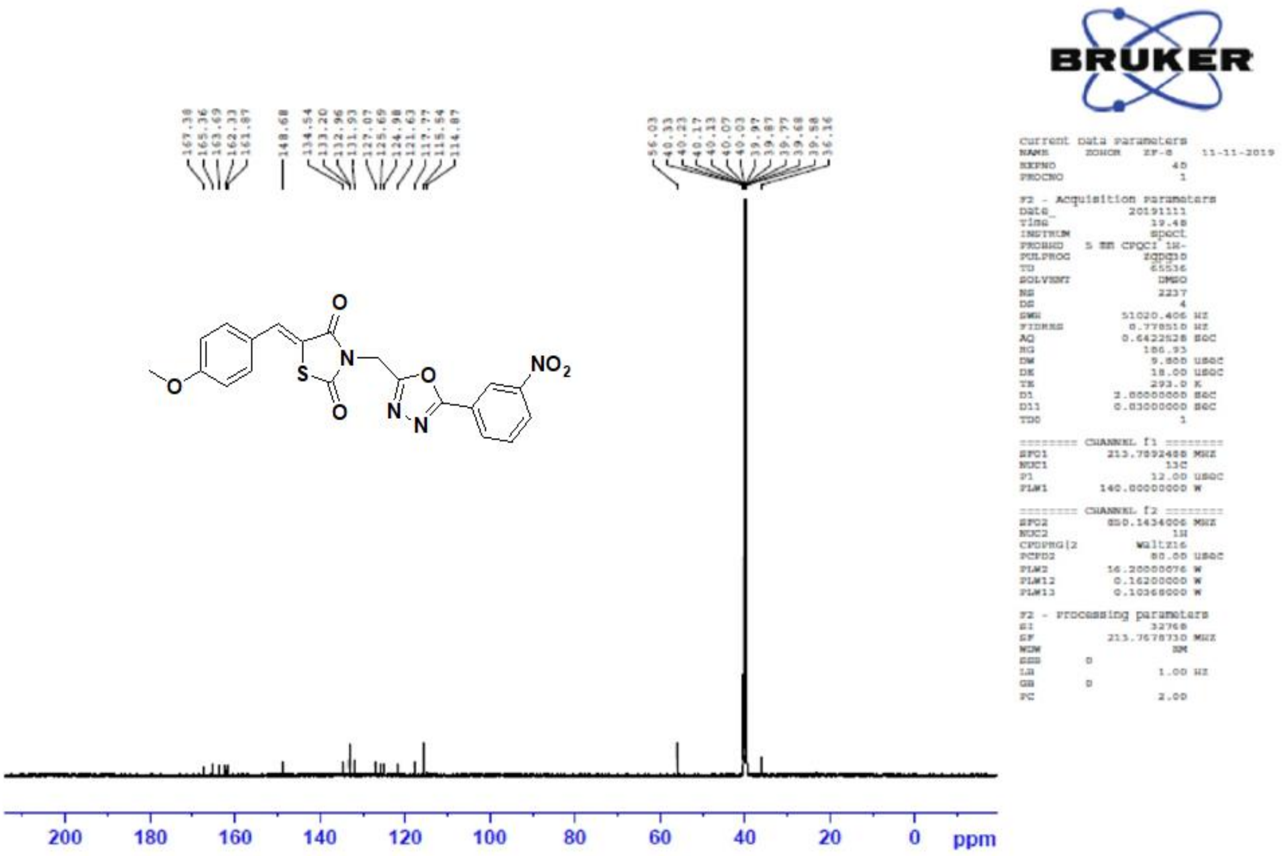

^{13}C NMR of compound 14

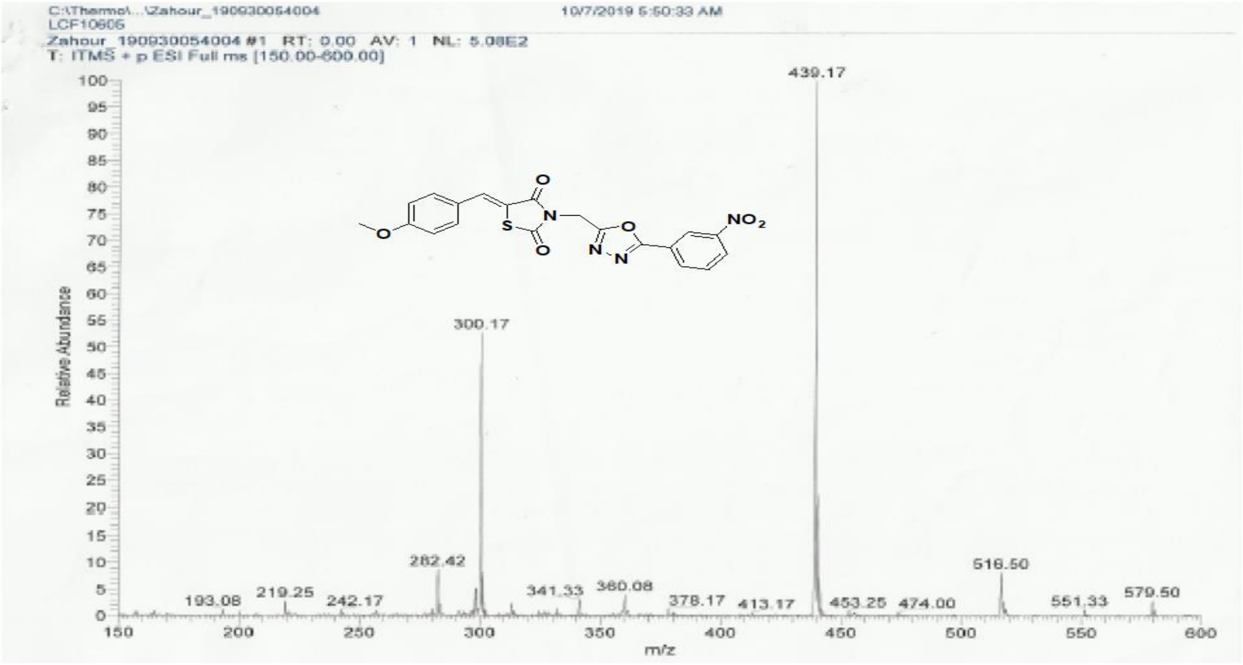

Mass of compound 14

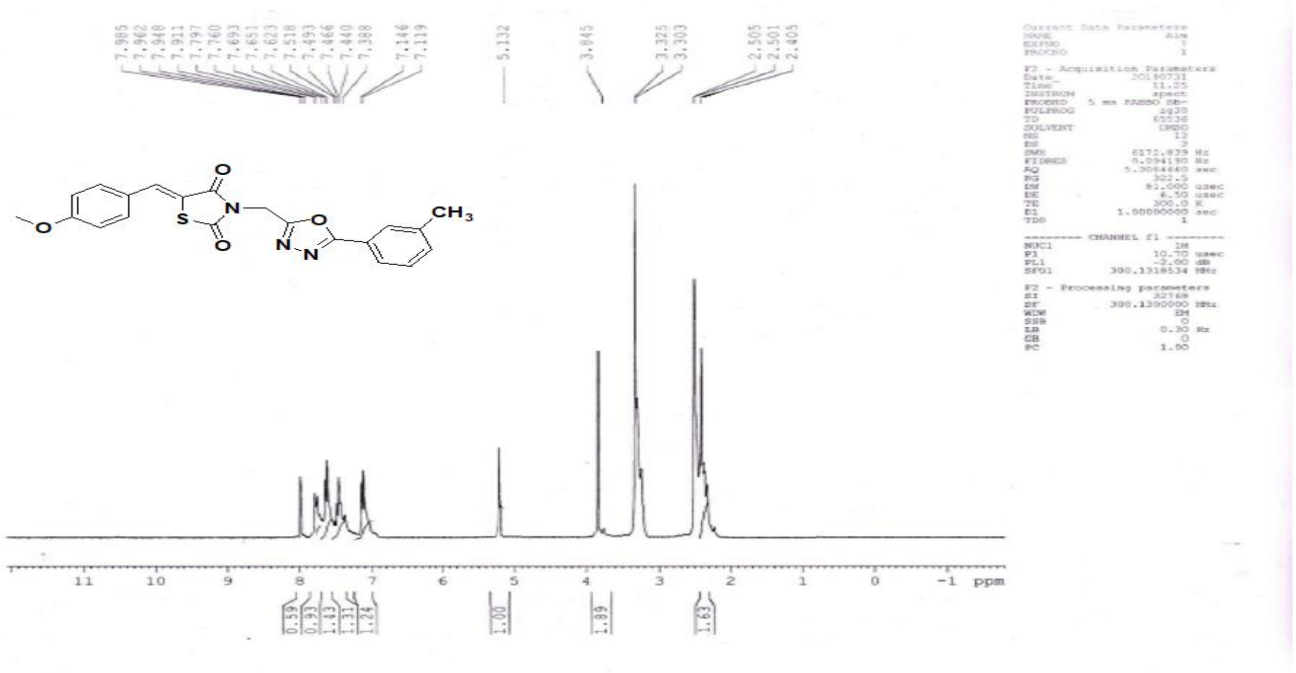

^{1}H NMR of compound 15

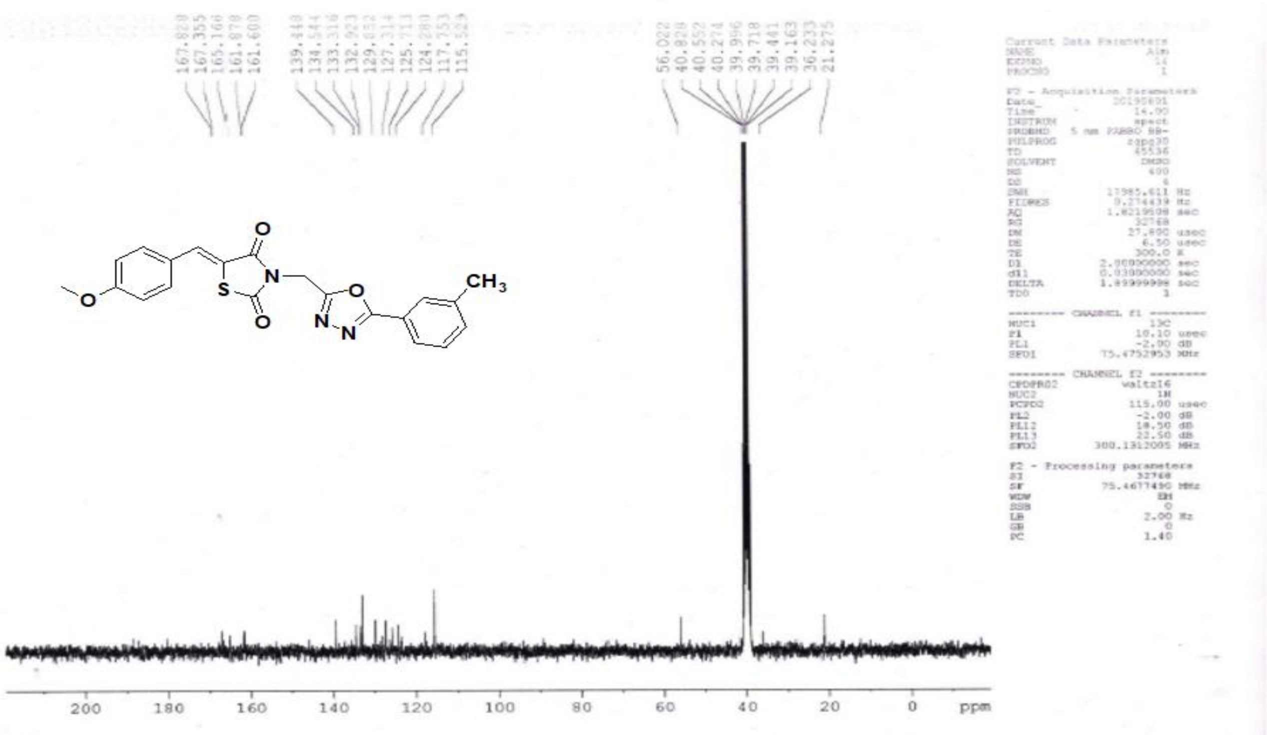

^{13}C NMR of compound 15

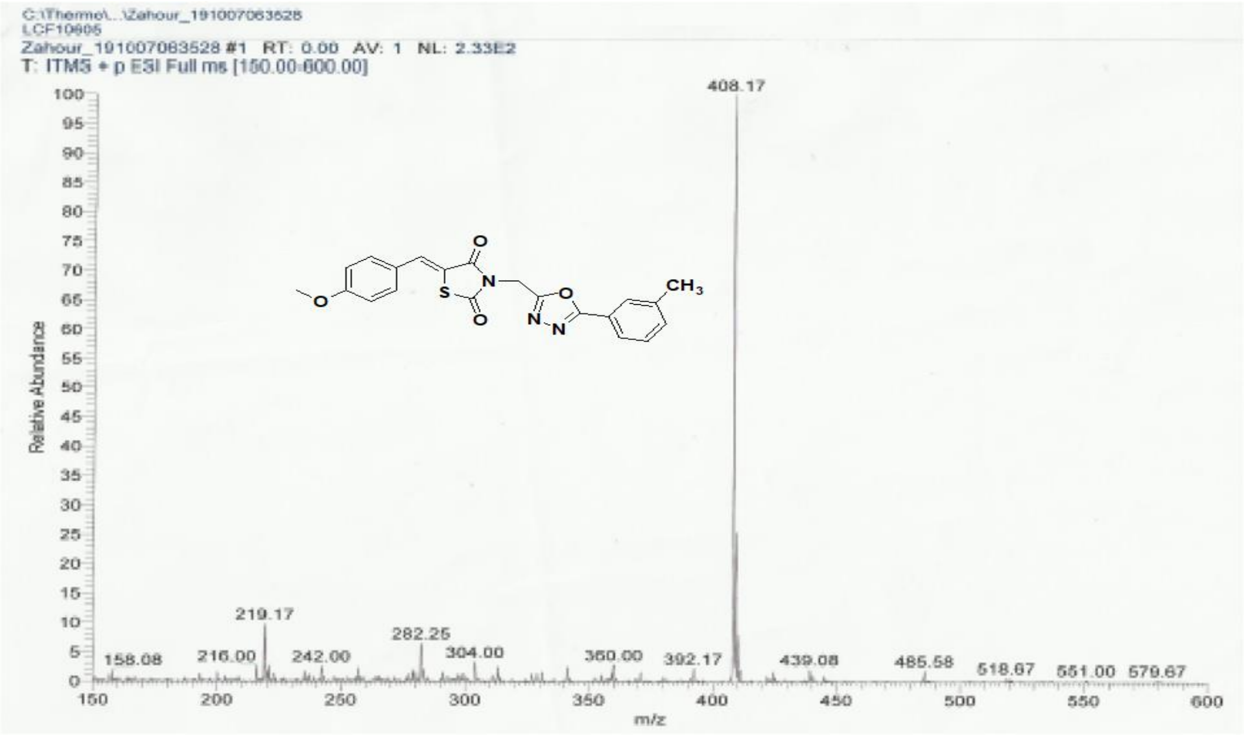

Mass of compound 15

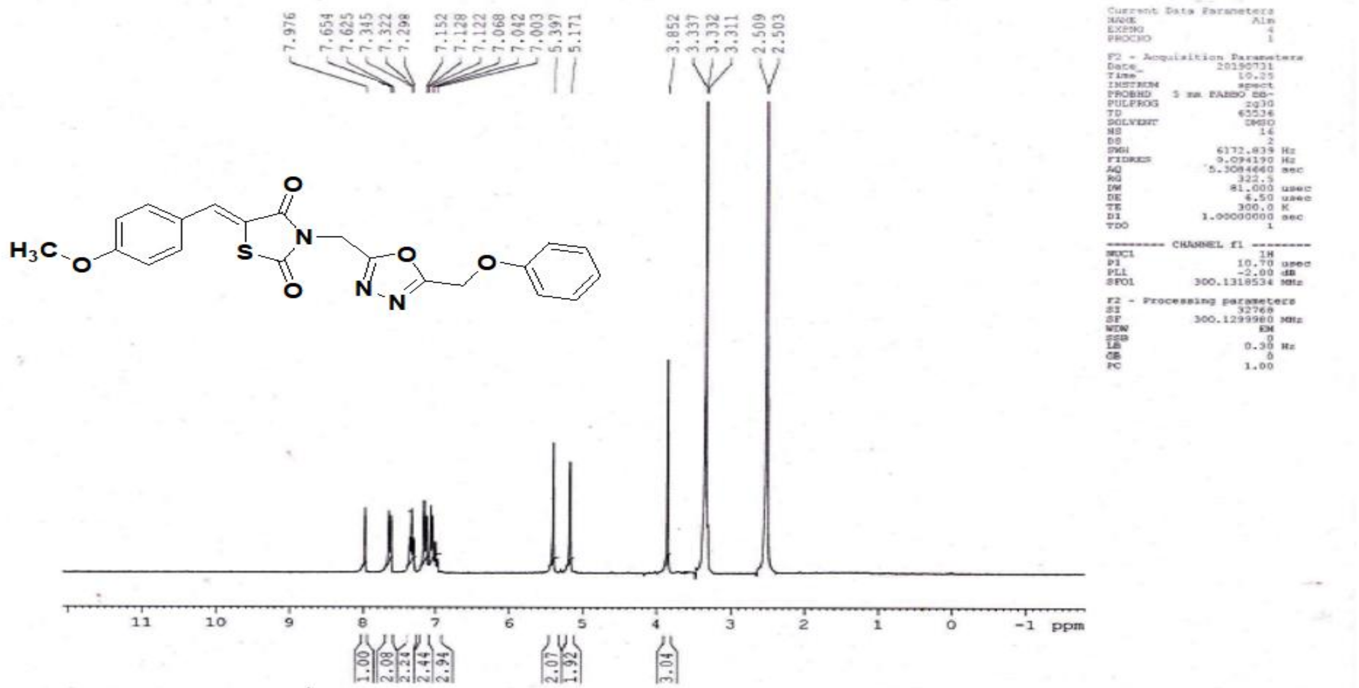

^{1}H NMR of compound 16

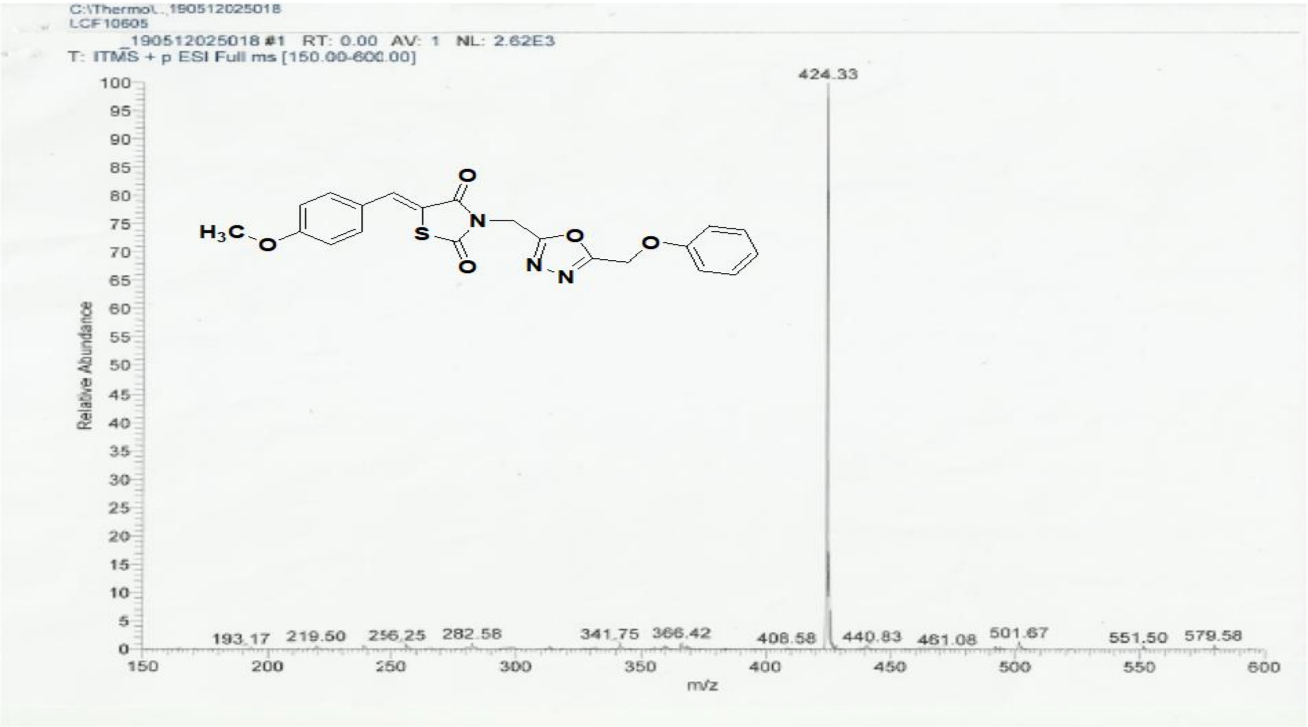

Mass of compound 16

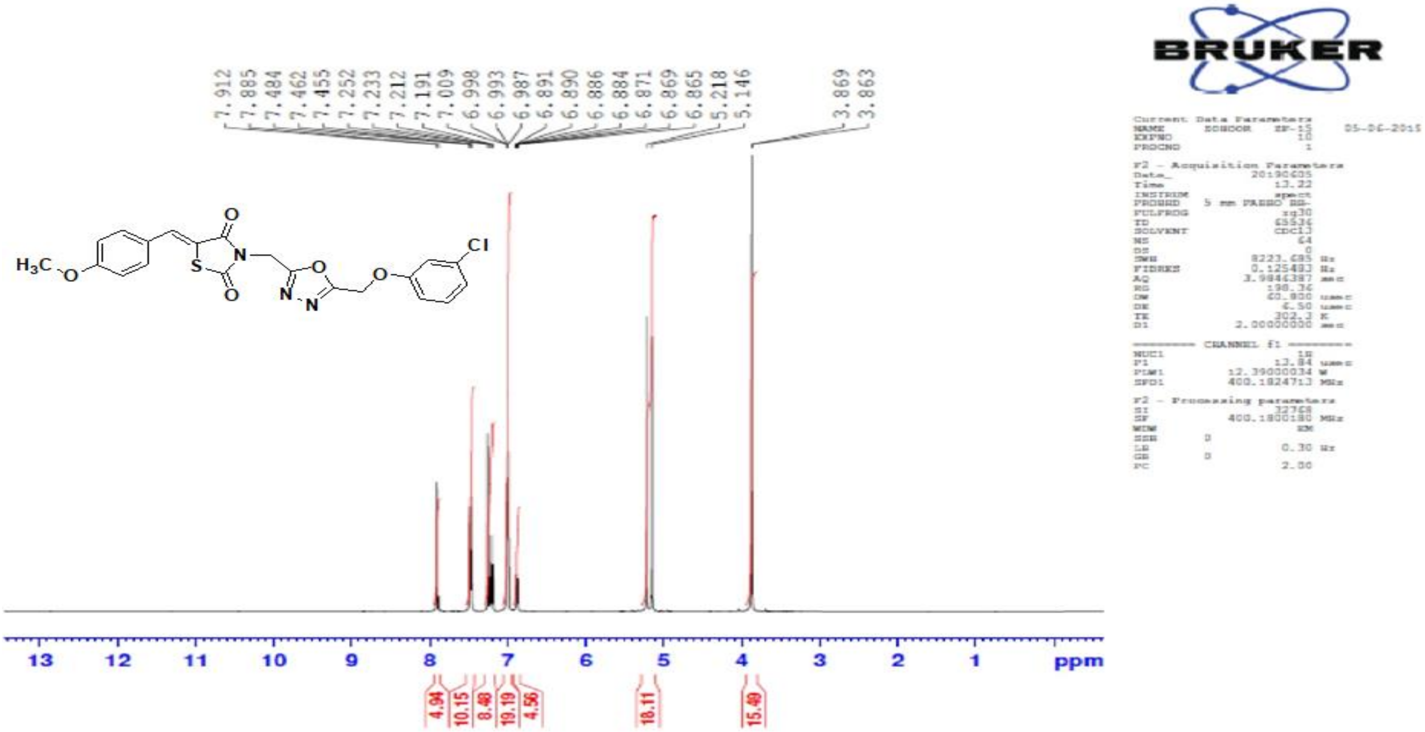

^{1}H NMR of compound 17

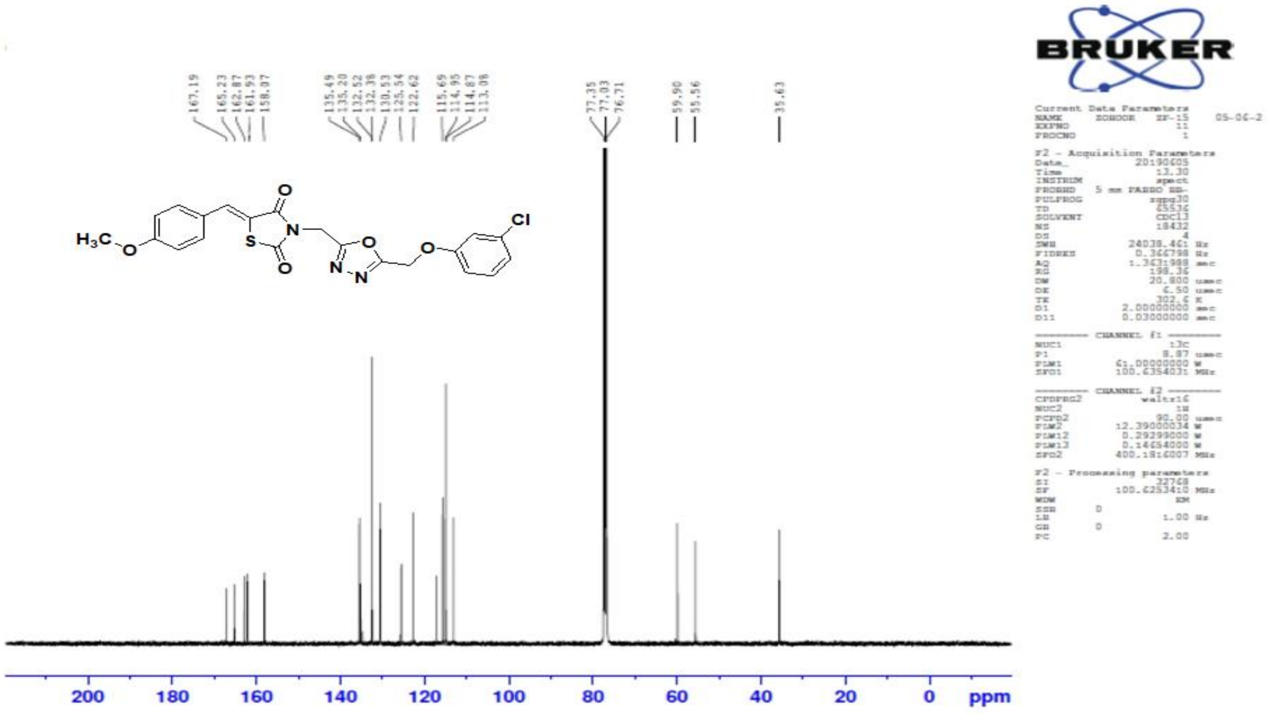

^{13}C NMR of compound 17

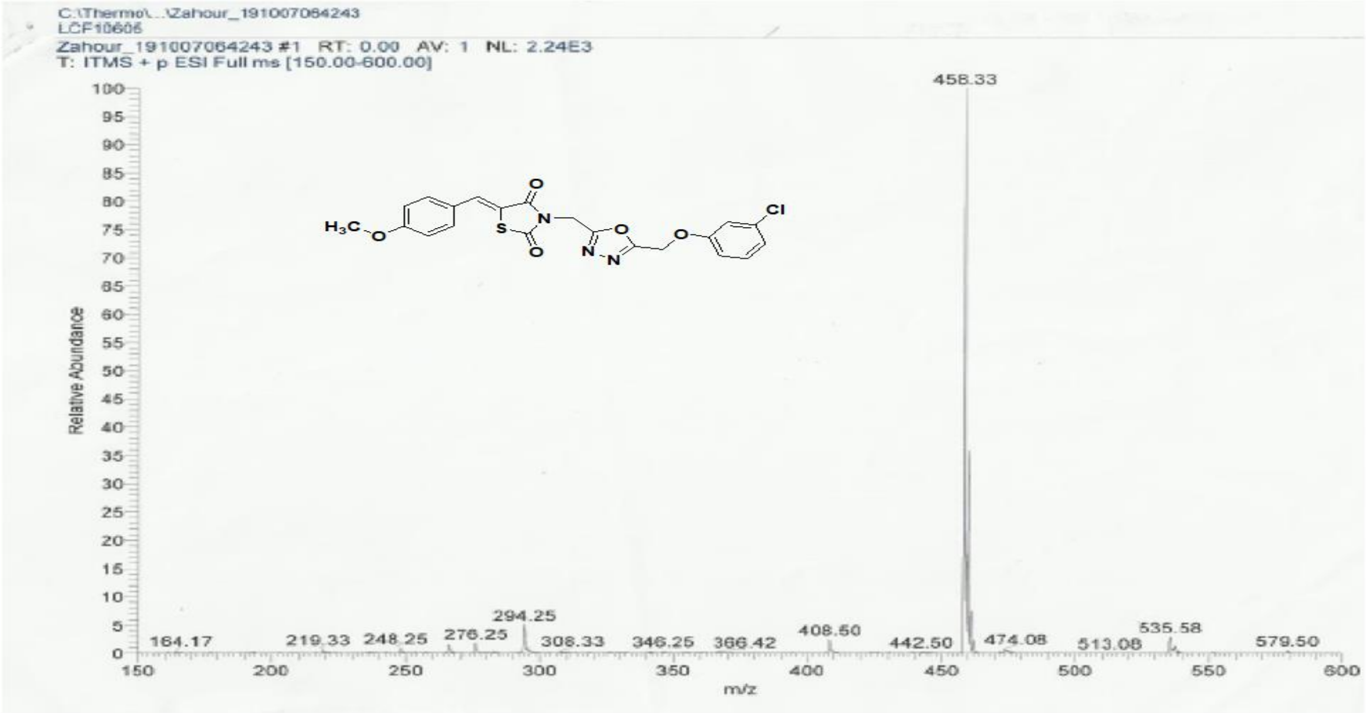

Mass of compound 17

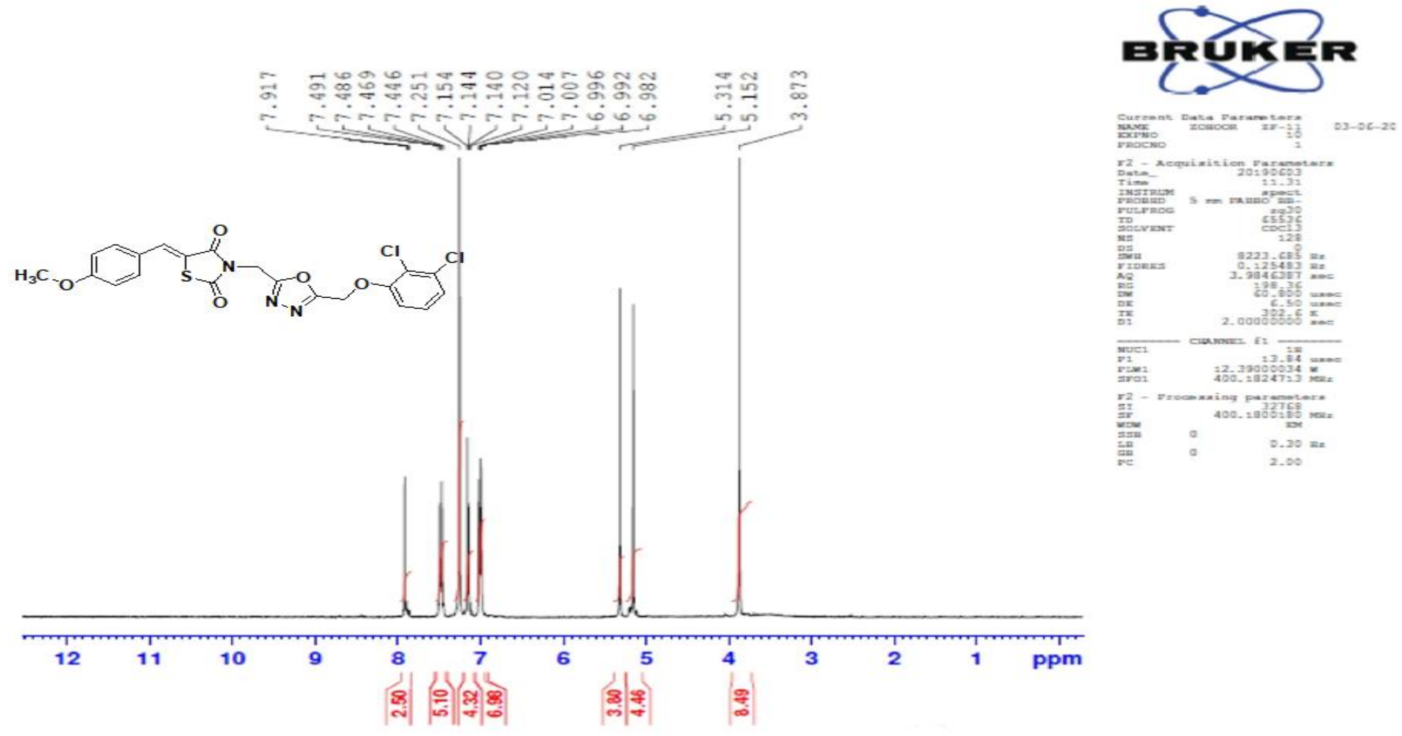

^{1}H NMR of compound 18

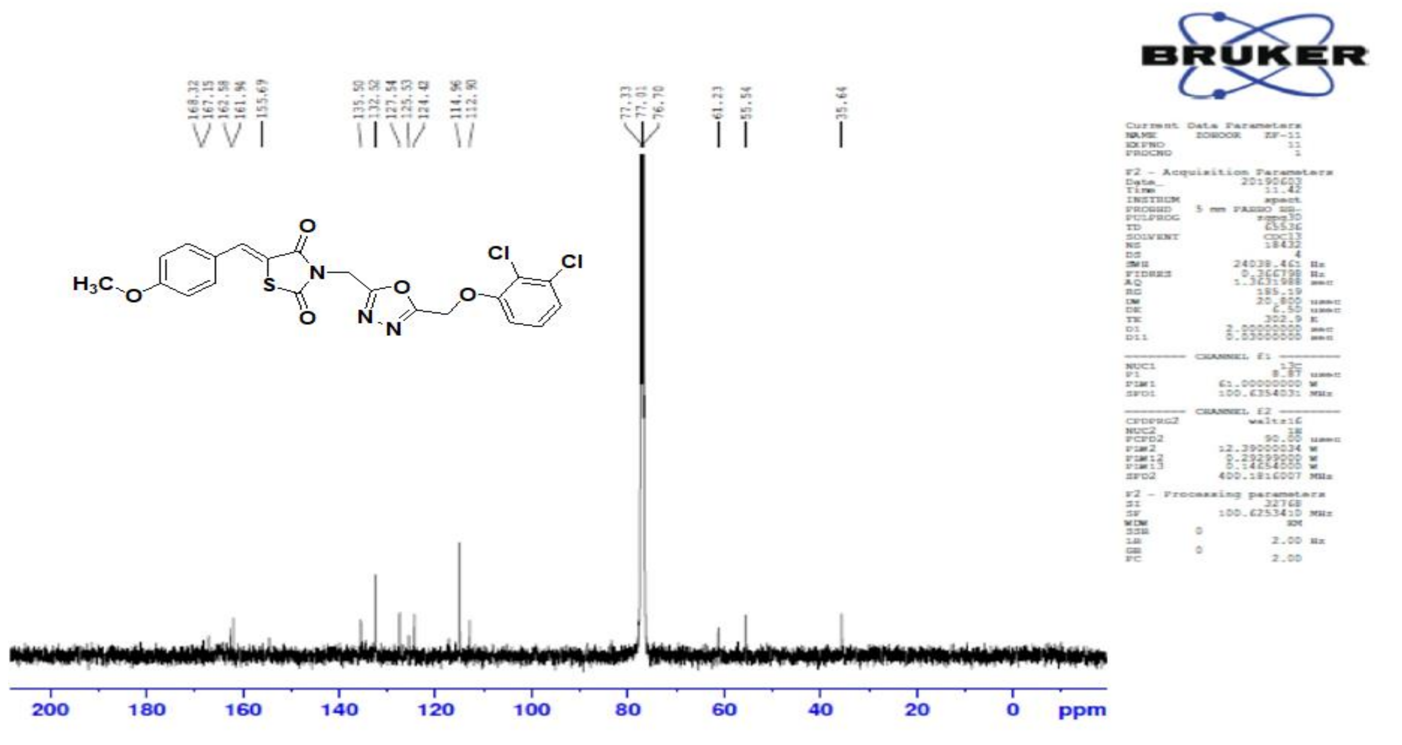

^{13}C NMR of compound 18

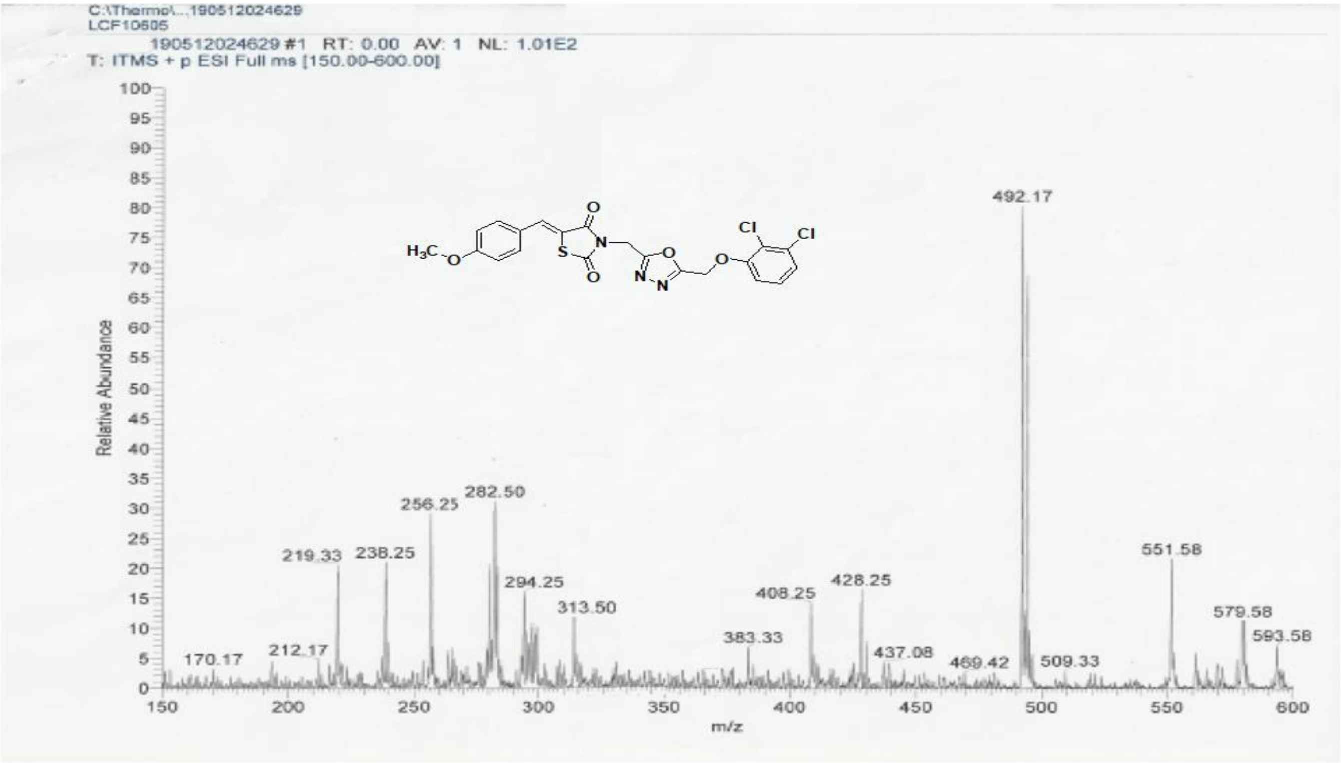

Mass of compound 18

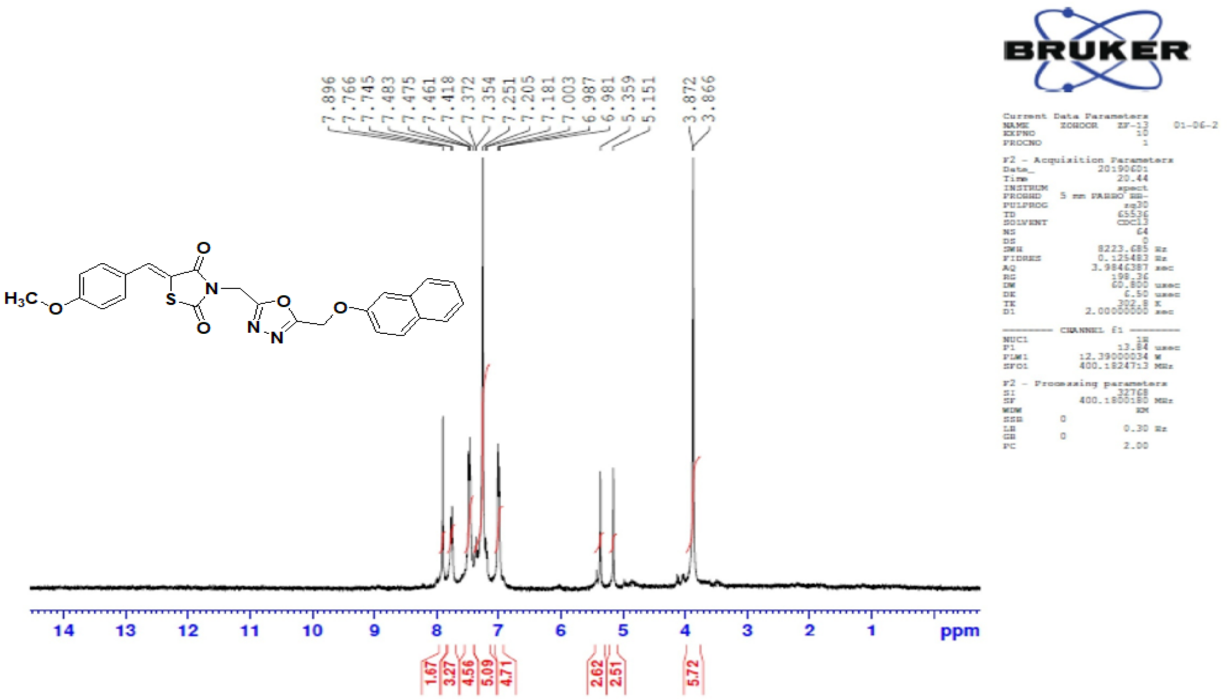

^{1}H NMR of compound 20

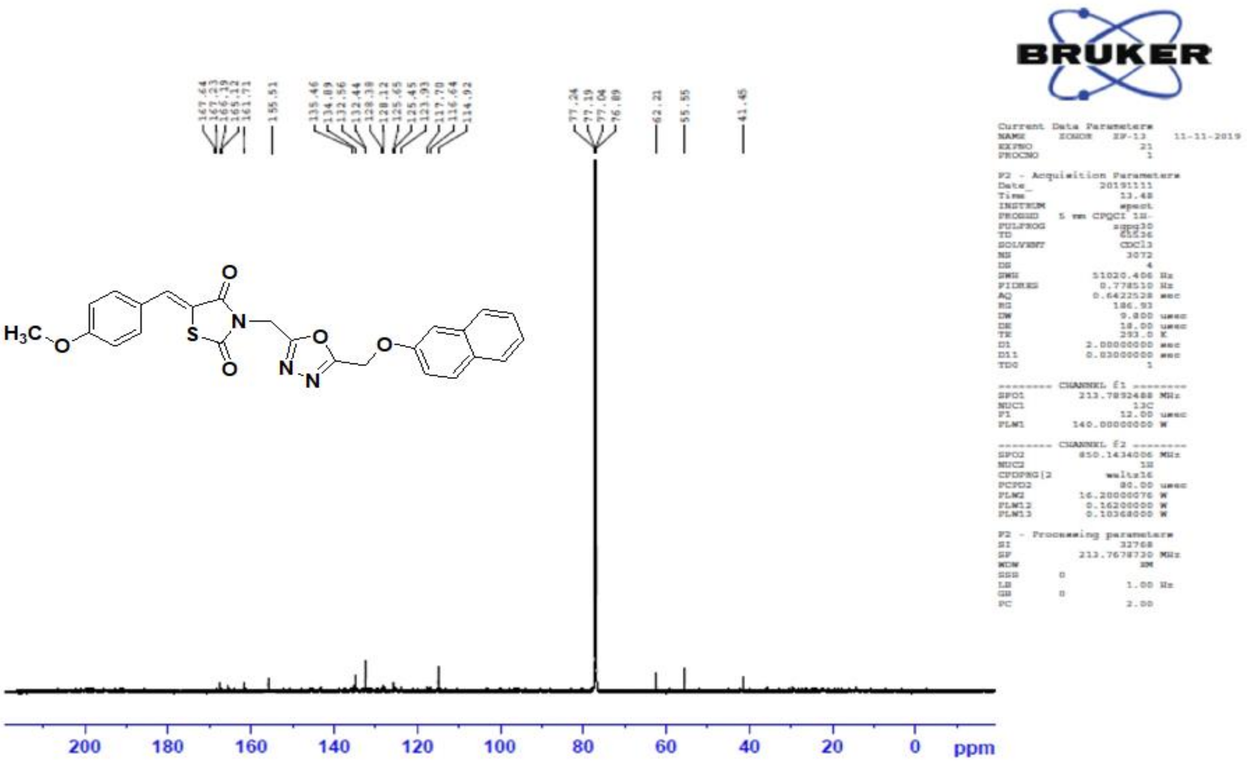

^{13}C NMR of compound 20

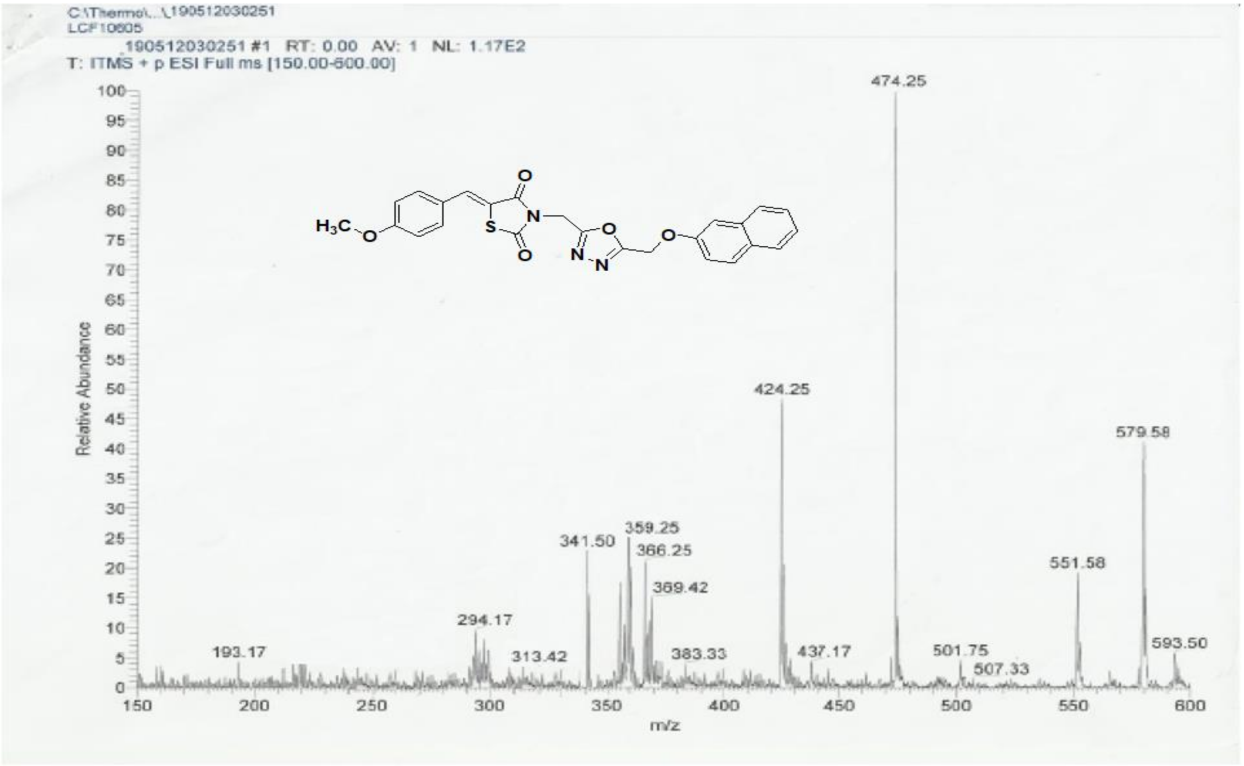

Mass of compound 20

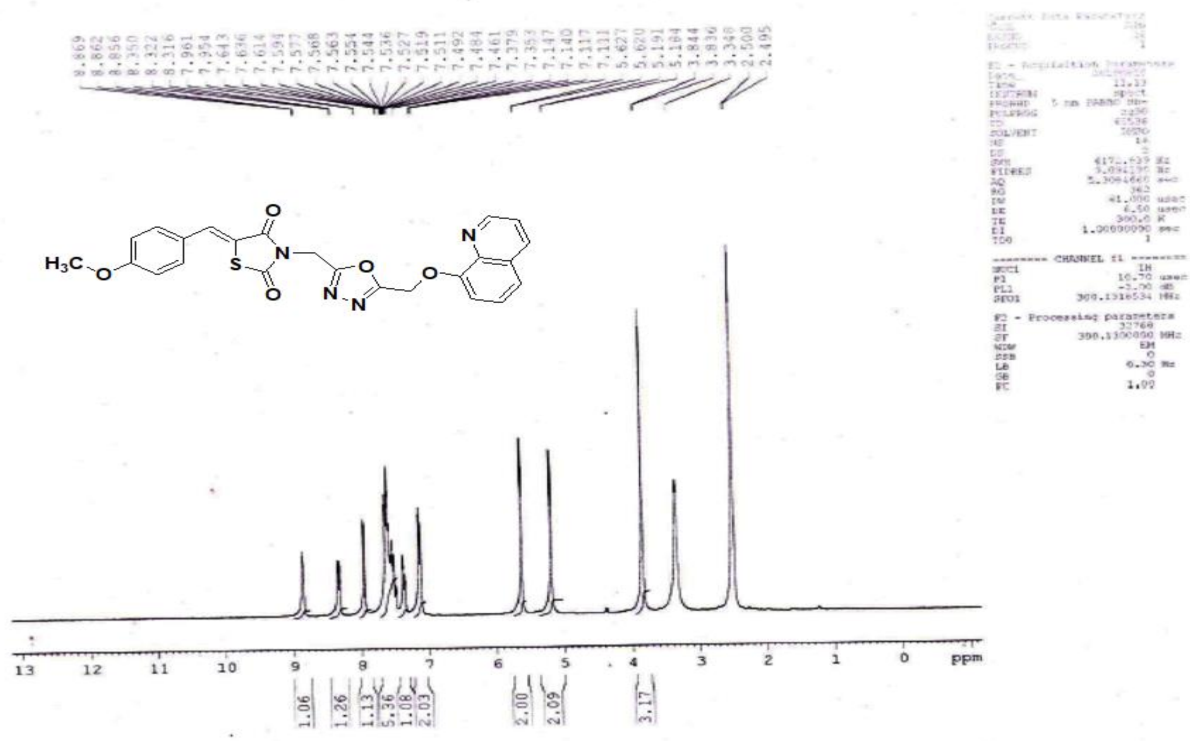

^{1}H NMR of compound 21

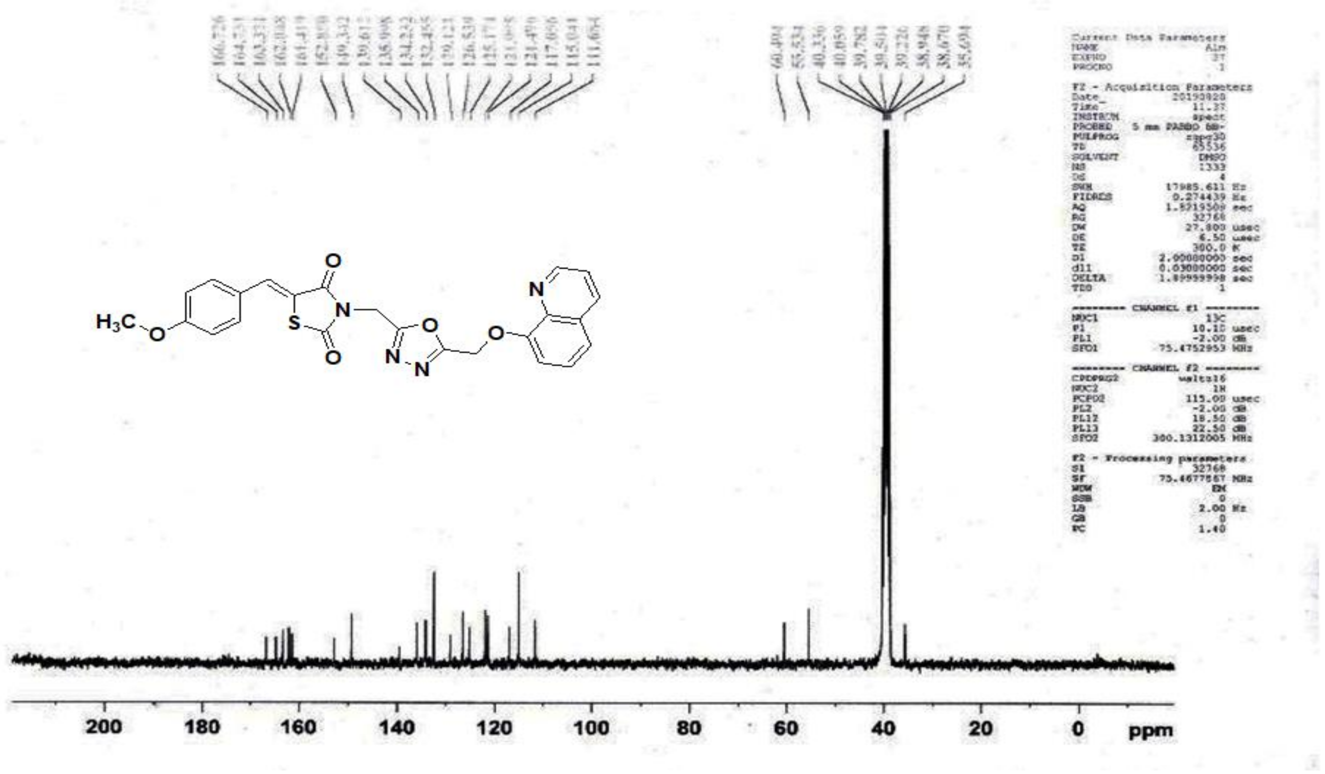

^{13}C NMR of compound 21

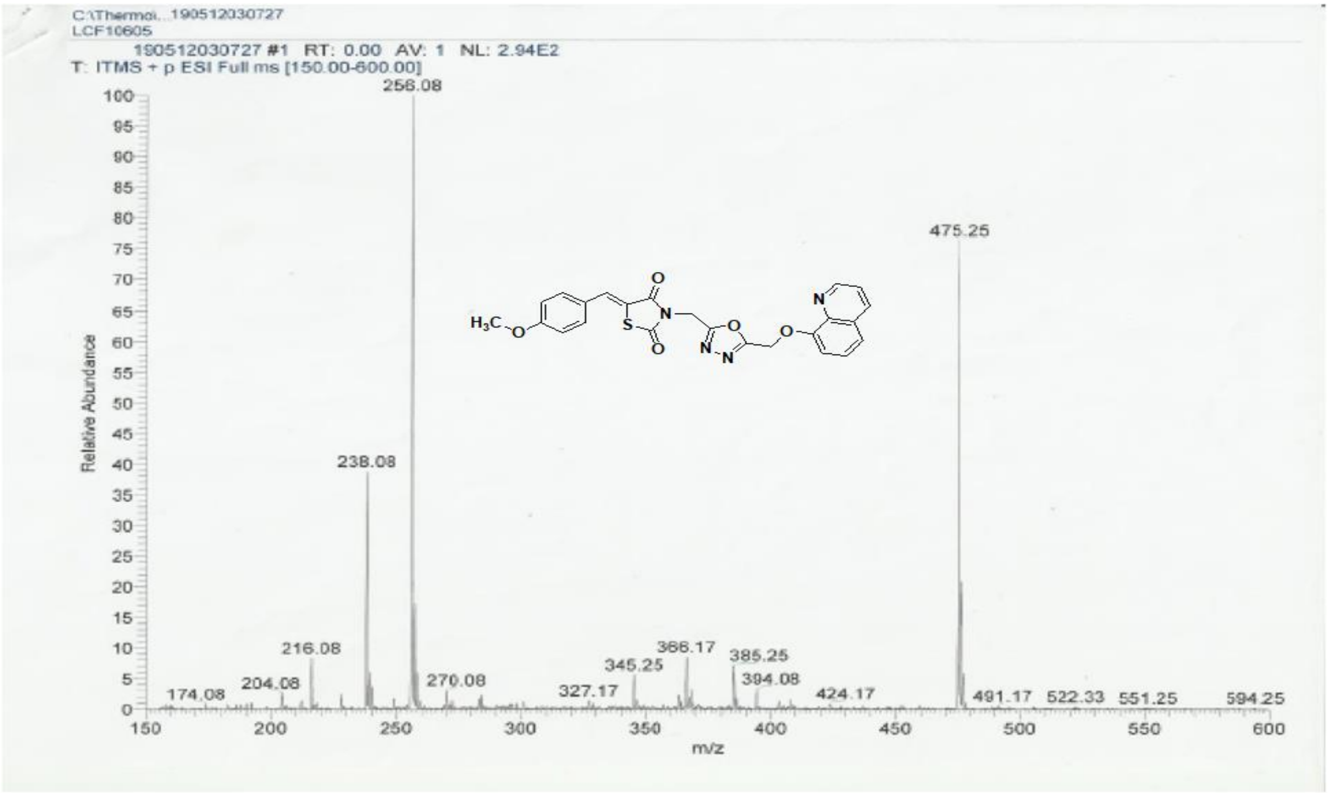

Mass of compound 21
